# Zebrafish ELL-associated factors Eaf1/2 modulate erythropoiesis *via* regulating *gata1a* expression and WNT signaling to facilitate hypoxia tolerance

**DOI:** 10.1186/s13619-022-00154-3

**Published:** 2023-04-01

**Authors:** WenYe Liu, ShuHui Lin, LingYa Li, ZhiPeng Tai, Jing-Xia Liu

**Affiliations:** College of Fisheries, Key Laboratory of Freshwater Animal Breeding, Ministry of Agriculture, Huazhong Agricultural University, 430070 Wuhan, China

**Keywords:** EAF1/2, *gata1a/scl/lmo2*, Erythropoiesis, H3K27me3, WNT/β-Catenin signaling, TCF4

## Abstract

EAF1 and EAF2, the eleven-nineteen lysine-rich leukemia (ELL)-associated factors which can assemble to the super elongation complex (AFF1/4, AF9/ENL, ELL, and P-TEFb), are reported to participate in RNA polymerase II to actively regulate a variety of biological processes, including leukemia and embryogenesis, but whether and how EAF1/2 function in hematopoietic system related hypoxia tolerance during embryogenesis remains unclear. Here, we unveiled that deletion of EAF1/2 (*eaf1*^−/−^ and *eaf2*^−/−^) caused reduction in hypoxia tolerance in zebrafish, leading to reduced erythropoiesis during hematopoietic processes. Meanwhile, *eaf1*^−/−^ and *eaf2*^−/−^ mutants showed significant reduction in the expression of key transcriptional regulators *scl*, *lmo2*, and *gata1a* in erythropoiesis at both 24 h post fertilization (hpf) and 72 hpf, with *gata1a* downregulated while *scl* and *lmo2* upregulated at 14 hpf. Mechanistically, *eaf1*^−/−^ and *eaf2*^−/−^ mutants exhibited significant changes in the expression of epigenetic modified histones, with a significant increase in the binding enrichment of modified histone H3K27me3 in *gata1a* promoter rather than *scl* and *lmo2* promoters. Additionally, *eaf1*^−/−^ and *eaf2*^−/−^ mutants exhibited a dynamic expression of canonical WNT/β-catenin signaling during erythropoiesis, with significant reduction in p-β-Catenin level and in the binding enrichment of both *scl* and *lmo2* promoters with the WNT transcriptional factor TCF4 at 24 hpf. These findings demonstrate an important role of Eaf1/2 in erythropoiesis in zebrafish and may have shed some light on regeneration medicine for anemia and related diseases and on molecular basis for fish economic or productive traits, such as growth, disease resistance, hypoxia tolerance, and so on.

**Supplementary Information:**

The online version contains supplementary material available at 10.1186/s13619-022-00154-3.

## Background

ELL-associated factors 1 and 2 (EAF1/2), a class of tumor suppressor genes interacting strongly with eleven-nineteen lysine-rich leukemia (ELL), can inhibit a variety of cancers in organisms, including leukemia and prostate cancer (Heydaran et al. 2021; Kenner 2014; Polak et al. 2003; Simone et al. 2003).

Several studies have revealed the functions and molecular characteristics of *EAF1/2* in *Arabidopsis* (Dabas et al. 2021; Scott et al. 1999), *Saccharomyces cerevisiae* (Laframboise and Baetz 2021; Sweta et al. 2021), *Caenorhabditis* (Cai et al. 2011) and mammalian cells (Kenner 2014), such as in cell growth (Dabas et al. 2018), cell immortalization (Dimartino et al. 2000) and in melanoma and leukemogenesis (Luo et al. 2001; Polak et al. 2003). It has been reported that *EAF1/2* can act as transcriptional suppressors to inhibit the TGF-β signaling pathway and WNT/β-catenin signaling in the formation of the three germ layers and the anterior and posterior pattern during early zebrafish embryogenesis (Liu et al. 2009; Liu et al. 2017; Liu et al. 2018; Liu et al. 2013). The level of p-β-Catenin indicates the undegenerated β-Catenin protein and WNT/β-catenin activities (Ahmadzadeh et al. 2016). Additionally, *EAF2* has been shown as a key factor mediating the androgen protection of DNA damage through Ku70/Ku80 in prostate cancer cells (Ai et al. 2017). Despite the ubiquitous expression of both genes, the deficiency of each EAF is associated with a particular clinical phenotype. However, to date, little is known about the association of *eaf1* and *eaf2* deficiency with molecular and physiological phenotypes in the hematopoietic system development.

Globin functions principally in oxygen-binding and delivery in various tissues and organs (Tian et al. 2017). The level of hemoglobin (Hb) in fish tends to increase under hypoxic conditions, which plays an important role in transporting oxygen and maintaining normal life activities (Fago 2017; Lee and Percy 2011; Lorenzo et al. 2014; Rahbar 1983; Roesner et al. 2008; Wawrowski et al. 2011; Xiao 2015). Meanwhile, studies have identified *EAF2* as a hypoxia response gene, which is specifically stimulated by HIF-1α rather than HIF-2α, enabling *EAF2* to protect cells against hypoxia-induced cell death and inhibit cellular uptake of glucose under hypoxic conditions (Chen et al. 2014; Pang et al. 2016; Xiao et al. 2009). To date, the roles of *eaf1* and *eaf2* in hypoxia tolerance by regulating erythropoiesis and the related underlying mechanisms remain almost entirely unclear.

Zebrafish (*Danio rerio*) is an ideal model for hematopoiesis research because its transparency greatly facilitates the visualization of blood cell formation (de Jong and Zon 2005; Paik and Zon 2010). Hematopoietic development in zebrafish has been considered to comprise two major overlapping hematopoiesis stages: “primitive” and “definitive” phases, both producing red blood cells (RBCs) (Davidson and Zon 2004; Paik and Zon 2010; Zhang et al. 2021). A sophisticated network of lineage-specific transcription factors *Scl/Tal1*, *Gata1*, and *Lmo2* was shown to function pivotally and essentially in erythrogenesis *via* regulating the expression of erythropoietic genes (Dooley et al. 2005; Ferreira et al. 2005; Galloway et al. 2005; Patterson et al. 2007). The gene *Gata2* may play a more important role in hematopoietic progenitor multi-potentiality (Tsai and Orkin 1997). Although the importance of these transcription factors has been demonstrated in cell-based *ex vivo* assays and knockout vertebrate models, the available information on the regulation of their expression is still limited.

The purpose of this study was to investigate the effects of EAF1/2 deficiency and the related molecular mechanism on erythropoiesis and hypoxia tolerance in zebrafish. *Eaf1*^*−/−*^ mutant with 5 bp deletion in exon1 has been successfully constructed in our laboratory (Liu et al. 2018). Here, we knocked out *eaf2* in zebrafish to generate homozygous mutants and tested the stress resistance of *eaf1*^*−/−*^ and *eaf2*^*−/−*^ mutants to hypoxia infection. The *eaf1*^−/−^ and *eaf2*^−/−^ mutants were found to exhibit increased sensitivity to hypoxia and defective erythropoiesis. Expression analysis of genes marking erythrocyte lineages and erythropoiesis revealed the reduced expression *scl*, *lmo2*, and especially *gata1a* during erythropoiesis in *eaf1*^*−/−*^ and *eaf2*^−/−^ larvae, suggesting they might be the primary endpoint contributors to defective erythropoiesis. Additionally, we investigated the mediators between *eaf1/2* and the three endpoint factors (*gata1a, scl* and *lmo2*) in erythropoiesis, and *eaf1/2* were shown to regulate erythropoiesis by modulating *gata1a* expression and WNT signaling to facilitate hypoxia tolerance.

## Results

### Loss of *eaf1* and *eaf2* leads to reduced hypoxia tolerance in zebrafish

Zebrafish carries two ELL-associated factors: *eaf1* and *eaf2* (Liu et al. 2009; Liu et al. 2013). The *eaf1-*deleted zebrafish has been constructed in our lab and reported recently (Fig. S1A1) (Liu et al. 2017; Liu et al. 2018), and *eaf2-*deleted zebrafish was constructed in this study (Fig. S1A2). When compared with wild-type (WT) siblings, *eaf1*^−/−^ and *eaf2*^*−/−*^mutants exhibited almost no Eaf1 protein and Eaf2 protein (Fig. S1B), respectively, which were also verified by qRT-PCR (Fig. S1C). Also, *eaf2*^−/−^ mutants exhibited overtly reduced *eaf2* transcripts (Fig. S1C). Meanwhile, *eaf1*^−/−^ and *eaf2*^−/−^ embryos were morphologically indistinguishable from WT embryos at 96 hpf (Fig. S1D). Additionally, homozygous *eaf1*^−/−^ and *eaf2*^−/−^ zebrafish could survive to adulthood in a viable and fertile state and were indistinguishable from WT adults (Fig. S1E).

**Fig. 1:**
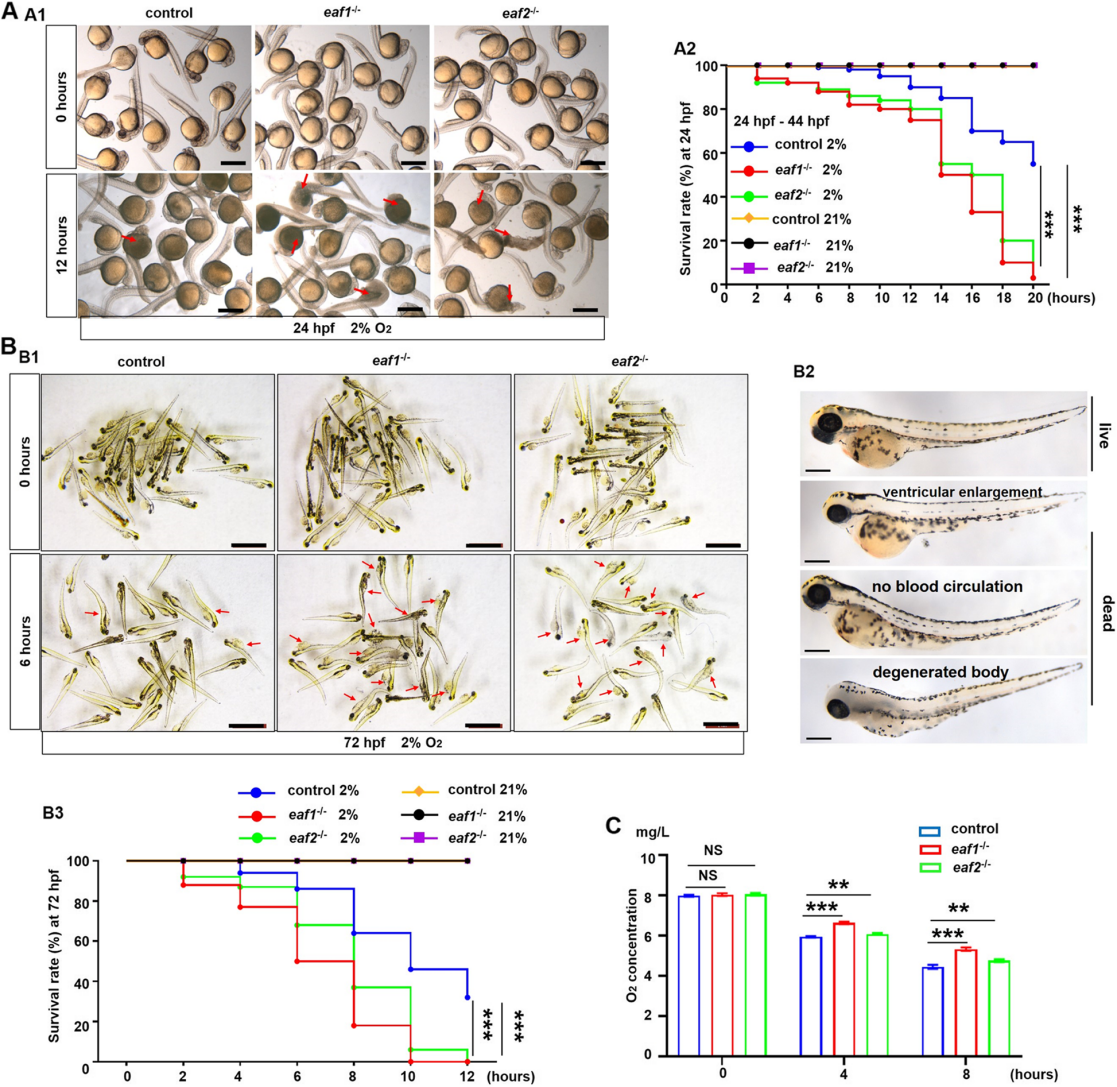
Effects of ***eaf1/2*** deficiency on hypoxia tolerance in zebrafish. **A** Representative images of *eaf1*^−/−^, *eaf2*^−/−^, and WT embryos exposed to hypoxia (2% O_2_) beginning at 24 hpf for 12 h and dead larvae were marked by red arrows (A1), and the survival rate curves of *eaf1*^−/−^, *eaf2*^−/−^ and wild-type (WT) embryos (A2). **B** Representative images of *eaf1*^−/−^, *eaf2*^−/−^, and WT larvae exposed to hypoxia beginning at 72 hpf for 12 h (B1), the representative images of living and dead larvae (marked by red arrows) (B2), and the survival rate curves of each group (B3). The oxygen concentration of the hypoxia workstation was adjusted to 2% before the experiment. The dead larvae were counted once every two hours, 30 embryos/larvae per group with three replicates. **C** Oxygen consumption rate was lower in *eaf1*^−/−^ and *eaf2*^−/−^ than in their WT siblings (6 mpf). Each experiment was repeated at least three times, with similar results for two or three replicates, and a representative result was shown. Data are mean ± SD. Hpf, hours post fertilization; dpf, days post fertilization; mpf, months post fertilization. B2, lateral view, anterior to the left. *P < .05, **P < .01, ***P < .001. NS, not significant. Scale bar = 2 mm (A1 and B1) and 100 μm (B2)

EAF2 has been reported to protect cells against hypoxia-induced cell death and inhibit cellular uptake of glucose under hypoxic conditions (Chen et al. 2014; Pang et al. 2016; Xiao et al. 2009), so we first tested the hypoxia tolerance of *eaf1*^−/−^ and *eaf2*^−/−^ mutants at 24 hpf, 72 hpf, and 6 mpf [6 months post-fertilization (mpf)] in this study. WT, *eaf1*^*−/−*^, and *eaf2*^−/−^ mutants at 24 hpf and 72 hpf were exposed to 2% O_2_, and after 12 and 20 h of hypoxic stress, both *eaf1*^−/−^ and *eaf2*^−/−^ larvae were all dead, with the mortality rate significantly (*P* < .001) higher in *eaf1*^−/−^ and *eaf2*^−/−^ mutants than in WT siblings during hypoxic stress (Figs. 1A-B). However, no significant difference was observed in the mortality rate between *eaf1*^−/−^/*eaf2*^−/−^ and WT embryos or larvae under normoxia (21% O_2_) (Figs. 1A-B, S2A).

Subsequently, we measured the hypoxia tolerance of adult *eaf1*^*−/−*^ and *eaf2*^−/−^ zebrafish by exposing each of *eaf1*^*−/−*^, *eaf2*^−/−^ and WT samples with a similar body weight (0.31 ± 0.04 g; mean ± SD) to hypoxia (5% O_2_, adjusted before experiment). During initial hypoxia stress, the *eaf1*^*−/−*^, *eaf2*^−/−^ and WT samples showed no obvious difference in locomotor behavior, and so on (Fig. S2B, Movie S1). However, after 30 min of hypoxia stress, compared to WT adult zebrafish, the *eaf1*^*−/−*^ and *eaf2*^−/−^ adult zebrafish showed the symptoms of dyspnea and swam to the surface for more oxygen. With increasing hypoxia time, the *eaf1*^*−/−*^ and *eaf2*^−/−^ adult zebrafish were dead or dying, whereas the WT adult zebrafish remained active (Fig. S2B, Movie S2).

Whether the difference between *eaf1*^*−/−*^*/eaf2*^−/−^ and WT zebrafish in hypoxia tolerance results from higher oxygen consumption in *eaf1*^*−/−*^ and *eaf2*^−/−^ was investigated. Unexpectedly, the oxygen consumption rate was even higher in the WT zebrafish than in the *eaf1*^*−/−*^ or *eaf2*^−/−^ zebrafish (Fig. 1C), indicating that the oxygen consumption is not the cause for the difference in hypoxia tolerance. The foregoing data suggested that disruption of *eaf1* and *eaf2* attenuated hypoxia tolerance in both larvae and adult zebrafish.

Additionally, whether the difference between *eaf1*^*−/−*^*/eaf2*^−/−^ and WT zebrafish in hypoxia tolerance results from the difference response of hypoxia genes. Expressions of hypoxia-inducible genes *hif1αb*, *hif2αb*, *hif3α*, *cited2*, *pai1* and *ldha* (Cai et al. 2018; Liu et al. 2016a) were tested and exhibited significantly decreased expression in *eaf1*^−/−^, *eaf2*^−/−^ embryos and larvae compared with their expressions in WT zebrafish under hypoxia (2% O_2_) (Figs. S3B-C), which is consistent with the down-regulated Hif-1a protein level in *eaf1*^−/−^ and *eaf2*^−/−^ mutants under hypoxic conditions (Fig. S3A).

### Disruption of *eaf1* and *eaf2* in zebrafish reduces erythrocytes

**Fig. 2:**
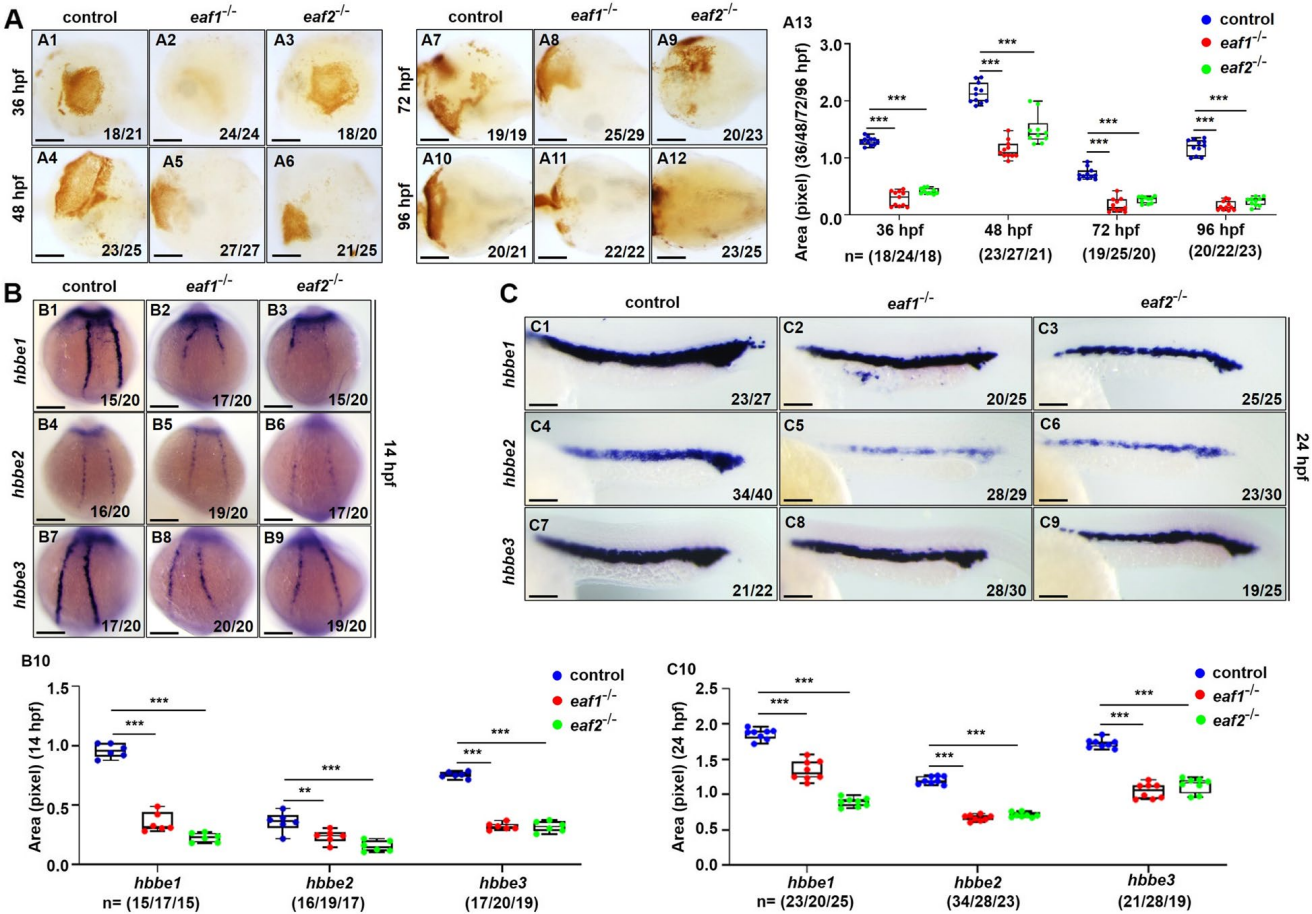
Effects of ***eaf1/2*** deficiency on erythrogenesis.** A** O-dianisidine staining analysis of erythrocytes at 36, 48, 72 and 96 hpf in both *eaf1*^−/−^ and *eaf2*^−/−^ embryos and larvae relative to WT (A1-A12). Statistical analysis of O-dianisidine staining results (A13). **B, C** WISH analysis of the expression of embryonic hemoglobin, *hbbe1/hbbe2/hbbe3* in *eaf1*^−/−^, *eaf2*^−/−^, and WT embryos at 14 hpf (B1-B9) and 24 hpf (C1-C9), respectively. The statistical analysis of WISH hemoglobin gene staining results for *eaf1*^−/−^, *eaf2*^−/−^, and WT embryos at 14 hpf (B10) and 24 hpf (C10), respectively. Each experiment was repeated at least three times, with similar results for two or three replicates, and a representative result was shown. Data are mean ± SD. A1-A12, ventral view, anterior to the left. B1-B9, dorsal view, anterior to the top. C1-C9, lateral view, anterior to the left. *P < .05, **P < .01, ***P < .001. NS, not significant. Scale bar = 75 μm (A1-A12) and 200 μm (B1- B9, C1- C9)

Under hypoxia stress, an effective strategy for fish to adapt to such stress is to increase the number of red blood cells and promote the oxygen-carrying capabilities of hemoglobin (Fago 2017; Roesner et al. 2008; Wawrowski et al. 2011). Given the importance of red blood cells (RBCs) in hypoxia tolerance, we assessed whether the loss of *eaf1* and *eaf2* in zebrafish can cause blood cell development defects. First, we used o-Dianisidine staining to measure the RBCs of *eaf1*^*−/−*^, *eaf2*^−/−^, and WT embryos at 36, 48, 72, and 96 hpf **(**Figs. 2A, S4A). Results showed that there were fewer o-Dianisidine-positive cells in the *eaf1*^*−/−*^ and *eaf2*^−/−^ embryos and larvae than that in the WT embryos and larvae (Figs. 2, S4A), suggesting that deletion of *eaf1* and *eaf2* significantly decreased the number of red blood cells during zebrafish embryogenesis.

Additionally, we investigated whether the reduction in erythrocytes in *eaf1*^*−/−*^ and *eaf2*^−/−^ embryos results from erythropoiesis defects by whole-mount in situ hybridization (WISH) analysis of *hbbe1*, *hbbe2*, and *hbbe3* transcripts in *eaf1*^*−/−*^ and *eaf2*^−/−^ zebrafish embryos at 14, 24, and 33 hpf (Figs. 2B, C; S4B). The expression levels of *hbbe1*, *hbbe2*, and *hbbe3* were significantly (*p* < .05) down-regulated in both *eaf1*^*−/−*^ and *eaf2*^−/−^ zebrafish relative to WT siblings, consistent with the above o-Dianisidine staining results. Furthermore, the flow cytometry analysis of hematopoietic cells from the adult whole kidney marrow (WKM) showed a significant (*P* < .05) decrease in erythrocytes with an increase in precursor cells in adult *eaf1*^−/−^ and *eaf2*^−/−^ mutants at 3 mpf, compared with those in WT zebrafish (Fig. S4C), suggesting that deletion of *eaf1* and *eaf2* significantly decreased the number of RBCs in adult zebrafish.

**Fig. 3:**
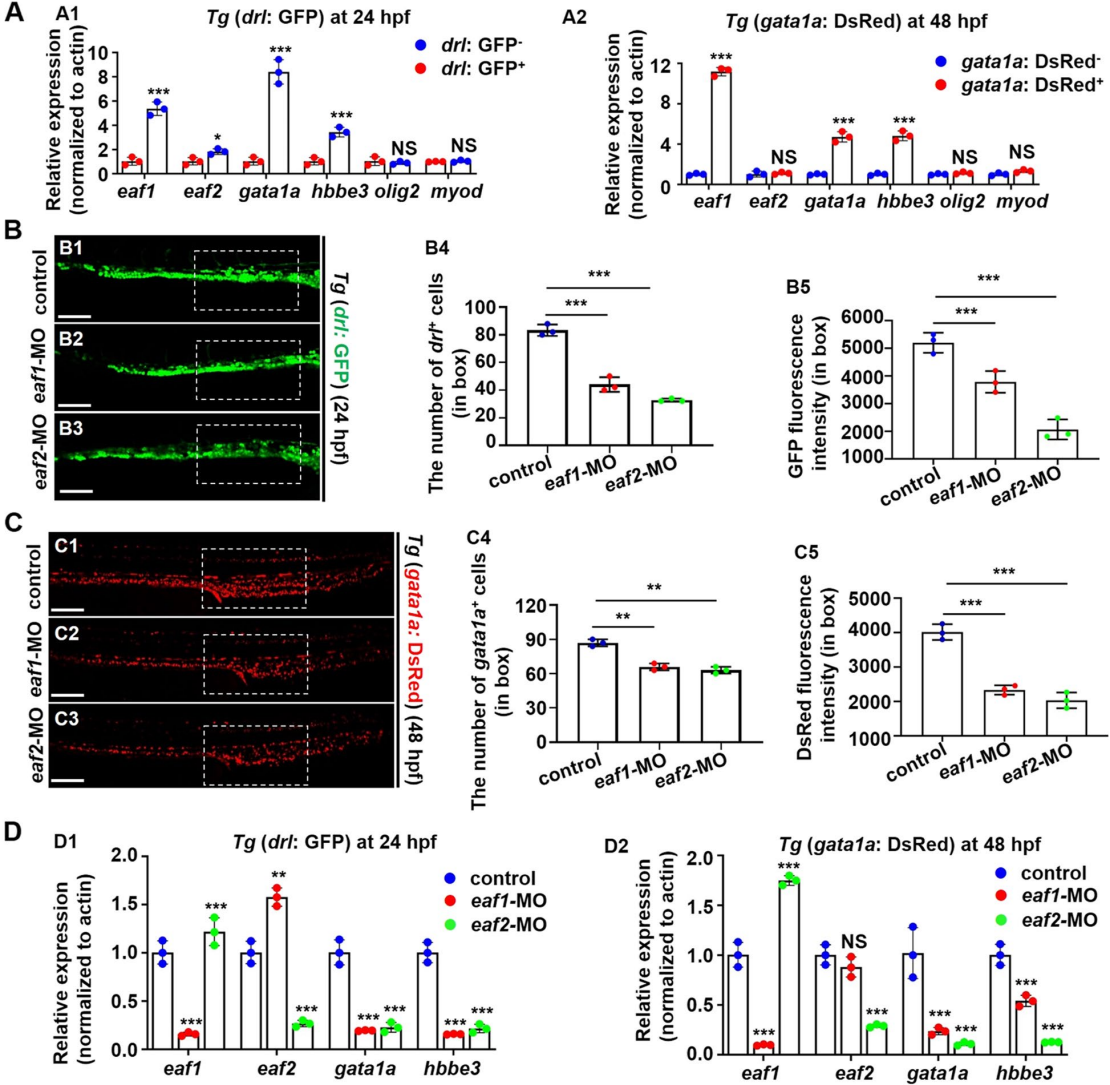
Effects of ***eaf1/2*** deficiency in erythrocytic-fluorescence transgenic fish.** A** Gene expressions, *eaf1*, *eaf2*, *gata1a*, *hbbe3*, *olig2* and *myod*, in *drl*^+^ or *gata1*^+^ cells collected from *Tg* (*drl*: GFP) (A1) and *Tg* (*gata1a*: DsRed) (A2) embryos, respectively. **B**,** C** Representative images of *Tg* (*drl*: GFP) (B1-B3) and *Tg* (*gata1a*: DsRed) embryos (C1-C3) injected with *eaf1*-MO or *eaf2*-MO at 24 hpf or 48 hpf, respectively, and quantification of the number of *drl*^+^ cells (B4) and *gata1a*^+^ cells (C4), and the GFP (B5) and DsRed (C5) fluorescence intensity in white box. **D** Gene expressions, *eaf1*, *eaf2*, *gata1a*, *hbbe3*, *olig2* and *myod*, in *drl*^+^ or *gata1a*^+^ cells collected from *Tg* (*drl*: GFP) (D1) and *Tg* (*gata1a*: DsRed) (D2) embryos injected with *eaf1*-MO or *eaf2*-MO, respectively. Each experiment was repeated at least three times with similar results for two or three replicates, and a representative result was shown. B1-B3, C1-C3, lateral view, anterior to the left. *P < .05, **P < .01, ***P < .001. NS, not significant. Scale bar = 100 μm (B1-B3, C1-C3)

The abundant expression of *gata1a* and *hbbe3* and low expression of the neural gene *olig2* and the muscle gene *myoD* were observed in *drl*^*+*^ (Fig. 3A1) and *gata1a*^*+*^ cells (Fig. 3A2) sorted from *Tg* (*drl*: GFP) (Prummel et al. 2019) and *Tg* (*gata1a*: DsRed) (Tai et al. 2022) embryos respectively, suggesting the RBC identity of both *drl*^*+*^ and *gata1a*^*+*^ cells. The abundant expression of *eaf1* and approximate expression of *eaf2* in both *drl*^*+*^ and *gata1a*^*+*^ cells (Fig. 3A), implied their potential involvement in RBC development. Antisense morpholinos targeting *eaf1* and *eaf2* (Liu et al. 2013) separately caused the marked reduction in the number of *drl*^*+*^ cells in *Tg* (*drl*: GFP) (Fig. 3B) and *gata1a*^*+*^ cells in *Tg* (*gata1a*: DsRed) (Fig. 3C), respectively, indicating that *eaf1* and *eaf2* are required for erythropoiesis. Deficiency of *eaf1* or *eaf2* caused markedly decreased expression of *gata1a* and *hbbe3* in both *drl*^*+*^ and *gata1a*^+^ cells (Fig. 3D). Meanwhile, embryos with knockdown of both *eaf1* and *eaf2* exhibited more reduced expression in *gata1a* and *hbbe3* compared with embryos with knockdown of either *eaf1* or *eaf2* (Fig. S5A). Additionally, in *eaf1*^−/−^ embryos at 24 hpf, the ectopic expression of *eaf2* mRNA partially restored the decrease of *hbbe3* (Fig. S5B) and *gata1a* (Fig. S5C) and *vice versa*, suggesting that *eaf1* and *eaf2* may function redundantly during zebrafish erythropoiesis.

### Disruption of zebrafish *eaf1* and *eaf2* affects the expression of erythropoiesis transcriptional factors

**Fig. 4:**
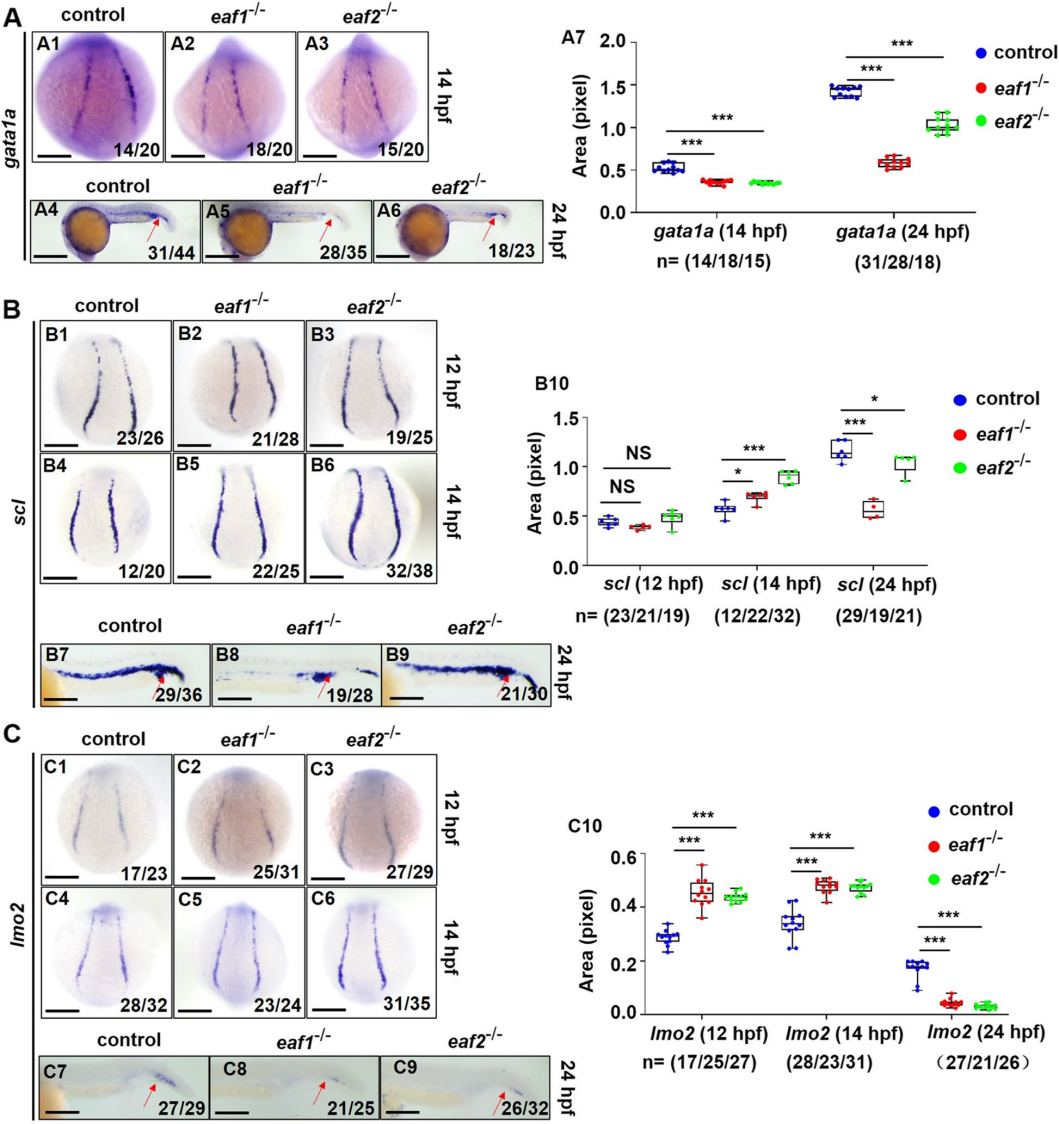
Effects of ***eaf1/2*** deficiency on the expression of erythrogenesis transcriptional factors.** A** WISH analysis of the expression of erythrogenesis transcriptional factor *gata1a* in *eaf1*^−/−^, *eaf2*^−/−^, and WT embryos at 14 hpf (A1-A3) and 24 hpf (A4-A6), and statistical analysis of *gata1a* staining results (A7). **B** WISH analysis of the expression of erythrogenesis transcriptional factor *scl* in *eaf1*^−/−^, *eaf2*^−/−^, and WT embryos (B1-B6) at 12/14 hpf and 24 hpf (B7-B9), and statistical analysis of *scl* staining results (B10). **C** WISH analysis of the expression of erythrogenesis transcriptional factor *lmo2* in *eaf1*^−/−^, *eaf2*^−/−^, and WT embryos at 12/14 hpf (C1-C6) and at 24 hpf (C7-C9). (C10) Statistical analysis of *lmo2* staining results. Each experiment was repeated at least three times, with similar results for two or three replicates, and a representative result was shown. Data are mean ± SD. A1-A3, B1-B6, C1-C6, dorsal view, anterior to the up; A4-A6, B7-B9, C7-C9, lateral view, anterior to the left. *P < .05, **P < .01, ***P < .001. NS, not significant. Scale bar = 75 μm (A1-A3, B1-B6, C1-C6), 200 μm (A4-A6), and 50 μm (B7-B9, C7-C9)

Each step of erythropoiesis is exquisitely regulated by specific factors, especially transcription factors and signaling molecules (Zhang et al. 2021).*Scl* (*tal1*) and *lmo2* are two primitive progenitor cell marker genes with pivotal functions in erythropoiesis (de Jong and Zon 2005). *Gata1* is essential for erythroid specification and formation, while *gata2* is essential for maintaining the hematopoietic progenitor pool (de Jong and Zon 2005). In this study, we used WISH to examine the expression of the aforementioned markers in *eaf1*^*−/−*^ and *eaf2*^−/−^ zebrafish embryos during erythropoiesis process. At 14 and 24 hpf, *gata1a* expression was clearly (*P* < .001) reduced in *eaf1*^*−/−*^ and *eaf2*^−/−^ embryos relative to WT embryos (Fig. 4A). At 14 hpf, *eaf1*^*−/−*^ and *eaf2*^−/−^ embryos showed significant (*P* < .05) up-regulation in the expression of both *scl* and *lmo2* relative to WT (Figs. 4B1-B6, C1-C6), in contrast to significant reduction in their expression at 24 hpf (Figs. 4B7-B9, C7-C9).

Additionally, we investigated whether the defective erythropoiesis is specific for hematopoietic system in *eaf1*^*−/−*^ and *eaf2*^−/−^ mutants during embryogenesis by WISH analysis of the expression of *gata2*, *fli1*, *flk1*, *runx1*, *c-myb*, *rag1*, and *myod*. No significant difference was detected in expression levels of *gata2* and *fli1* between *eaf1*^*−/−*^/*eaf2*^−/−^ and WT embryos at 14 hpf (Fig. S6A-B). The expression of vascular cell markers *flk1* and *fli1* remained unchanged in *eaf1*^*−/−*^ and *eaf2*^−/−^ embryos relative to WT embryos at 24 hpf (Fig. S6C). Meanwhile, the mesoderm progenitor *myod* also remained unchanged in the mutants at 24 hpf (Fig. S6D), indicating that overall embryogenesis was not influenced by disruption of *eaf1* and *eaf2*.

Meanwhile, the hematopoietic stem and progenitor cell (HSPC) markers (*runx1* and *c-myb*) showed an overtly (*P* < .001) upregulated expression in *eaf1*^*−/−*^ and *eaf2*^−/−^ embryos relative to WT embryos at both 14 and 33 hpf (Figs. S7A-B). However, the *rag1* (lymphoid marker), *gata1a* and *lmo2* exhibited reduced (*P* < .05) expression in *eaf1*^*−/−*^ and *eaf2*^−/−^ larvae relative to WT larvae at 5 days post fertilization (dpf) (Fig. S7C) or 72 hpf (Fig. S7D**)**, suggesting the RBC differentiation defects in the mutants.

These results indicated that *eaf1/2* deletion affects the expression of erythroid transcription factors *scl*, *lmo2* and *gata1a* in zebrafish during primitive and definitive erythropoiesis, reducing the number of erythrocytes and downregulating the terminal differentiation of erythroid cells, resulting in the increase of hematopoietic progenitor cell pool.

### *Eaf1* and *eaf2* regulate *scl* and *lmo2* transcription by modulating canonical WNT/β-catenin signaling in a developmental stage specific manner

**Fig. 5:**
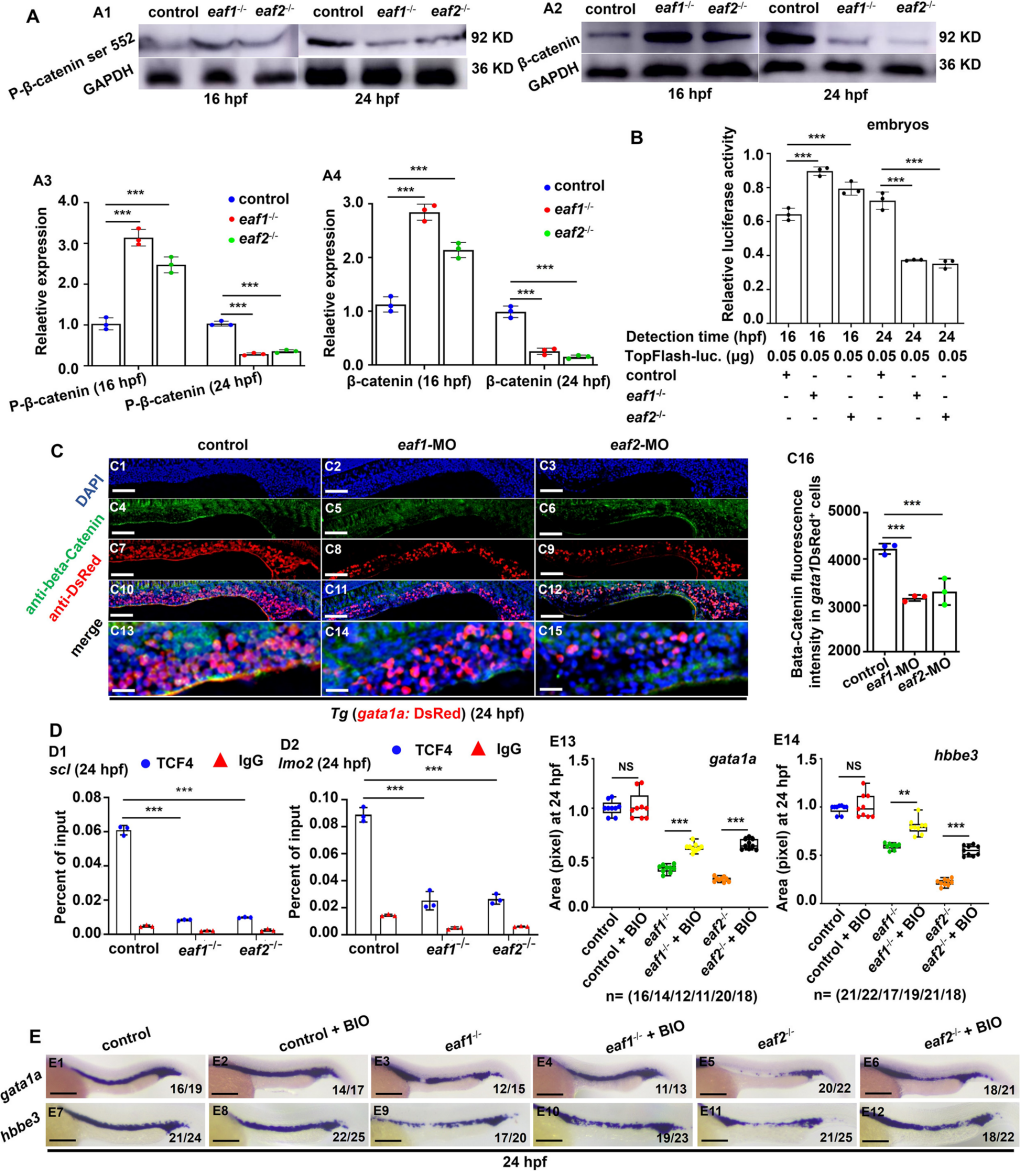
Effects of ***eaf1/2*** deficiency on WNT/β-catenin signaling during fish erythrogenesis. **A** Protein levels of P-β-catenin ser 552 and β-catenin in *eaf1*^−/−^, *eaf2*^−/−^, and WT embryos at 16 hpf and 24 hpf (A1-A2), respectively, and quantitative analysis of protein level in each sample (A3-A4). **B** Endogenous WNT/β-catenin signaling activities in *eaf1*^−/−^, *eaf2*^−/−^, and WT embryos at 16 hpf and 24 hpf, respectively. One-cell stage embryos were injected with TopFlash (as a reporter) and TK-renilla (as an internal control) together, and the injected embryos were collected for assays at 16 hpf and 24 hpf, respectively. **C** Double staining of *gata1a*DsRed^+^ and β-Catetin, in the control and embryos injected with *eaf1*-MO or *eaf2*-MO at 24 hpf (C1-C15), and quantification of β-Catenin immunofluorescence intensities in *gata1a*DsRed^+^ cells (C16), and C13-C15 show the magnified views of C10-C12, respectively. **D** Chromatin immunoprecipitation (ChIP) analysis of the binding enrichment of protein TCF4 on the promoter of gene *scl* (D1) and gene *lmo2* (D2) in *eaf1*^−/−^ and *eaf2*^−/−^ embryonic cells at 24 hpf, respectively, with anti- IgG used as a negative control. **E** WISH analysis of the expression of *gata1a* and *hbbe3* in *eaf1*^−/−^, *eaf2*^−/−^, WT embryos and the corresponding groups treated with Wnt activator BIO at 24 hpf (E1-E12), and statistical analysis of WISH results (E13, E14). Each experiment was repeated at least three times, with similar results for two or three replicates, and a representative result was shown. Data are mean ± SD. C1-C15, E1-E12, lateral view, anterior to the left. *P < .05, **P < .01, ***P < .001. NS, not significant. Scale bar = 200 μm (E1-E12), 100 μm (C1-C12), and 50 μm (C13-C15)

Eaf1/2 were unveiled to regulate the anterior-posterior pattern of zebrafish axis by modulating WNT/β-catenin signaling (Liu et al. 2018; Liu et al. 2013), which was shown to be involved in the hematopoietic developmental processes, such as early hematopoiesis and erythroid specification (Tarafdar et al. 2013; Wilusz and Majka 2008). In this study, we used WISH to examine the expression of Wnt signaling ligands *wnt3* and *wnt16*, WNT-activated receptor (*fzd2*), and *axin2* (a target of WNT/β-catenin signaling) in *eaf1*^*−/−*^ and *eaf2*^−/−^ embryos or larvae at 16 hpf, 24 hpf, or 72 hpf, respectively. At 16 hpf, *axin2*, *wnt16*, and *fzd2* all showed an increased expression (*P* < .001) in *eaf1/2* mutants relative to WT embryos, and at 24 hpf, *wnt16* and *fzd2* maintained the upregulated expression, while WNT/β-catenin activity indicator *axin2* was obviously down-regulated (*P* < .001) in *eaf1/2* mutants (Figs. S8A-B), consistent with tendency of β-Catenin and Phospho-β-Catenin (Ser552) (P-β-Catenin-Ser552) protein levels in e*af1*^*−/−*^ and *eaf2*^−/−^ embryos (Fig. 5A). Additionally, the dynamic WNT/β-catenin activities were observed in e*af1*^*−/−*^ and *eaf2*^−/−^ embryos **(**Fig. 5B**)** using 8×TopFlash luciferase reporter assays [which is commonly used to evaluate the transcriptional activity of β-Catenin (Aoki et al. 1999; Liu et al. 2017; Playford et al. 2000)], with significantly increased TopFlash luciferase activities at 16 hpf while reduced activities at 24 hpf in the mutants. Similarly, Top-GFP expression was also weakened in the brain (Fig. S7D) and spinal cord (Fig. S8E) in the *Tg* (*top*: dGFP) embryos with knockdown of either *eaf1* or *eaf2* at 24 hpf. In *Tg* (*gata1a*: DsRed) embryos, knockdown of either *eaf1* or *eaf2* resulted in the decrease of *gata1a*^+^ cells (RBCs) with decreasing β-Catenin level in the RBCs at 24 hpf (Figs. 5C, S9A). Meanwhile, *eaf1*^*−/−*^, *eaf2*^−/−^, and WT embryos showed no significant difference in the expression level of *wnt3* at both 16 hpf and 24 hpf (Fig. S8A-B). Additionally, at 72 hpf, deficiency of *eaf1* or *eaf2* resulted in significant decrease in expression of *axin2* (Fig. S8C) and β-Catenin protein in RBCs (*gata1a*^+^ cells) (Fig. S9B). The decreased expression of *gata1a* and *hbbe3* could be effectively rescued by treatment of Wnt agonist BIO at 24 hpf (Fig. 5E).

The transcriptional factor TCF4 (T-cell factor 4) in WNT/β-catenin signaling was reported to bind the promoters of genes *scl* and *lmo2* and regulate their expression (Liu et al. 2016b; Sturgeon et al. 2014). In this study, loss of *eaf1* or *eaf2* caused significant down-regulation of *tcf4* in *drl*^+^ and g*ata1a*^+^ erythrocytes (Fig. S9C). Meanwhile, we used chromatin immunoprecipitation-qPCR (ChIP-qPCR) to analyze the binding enrichment of protein TCF4 on *scl* and *lmo2* promoters in this study. Compared with the control, *eaf1*^*−/−*^ and *eaf2*^−/−^ embryos showed reduction (*P* < .001) in the binding enrichment of TCF4, the major endpoint mediator of the WNT signaling, in both *scl* and *lmo2* promoters at 24 hpf (Fig. 5D). These results suggested that disruption of zebrafish *eaf1* and *eaf2* may reduce TCF4 enrichment in the *scl* and *lmo2* promoters, thus down-regulating WNT/β-catenin signaling at 24 hpf.

### *Eaf1* and *eaf2* modulate *gata1a* transcription through an epigenetic modified mechanism

**Fig. 6:**
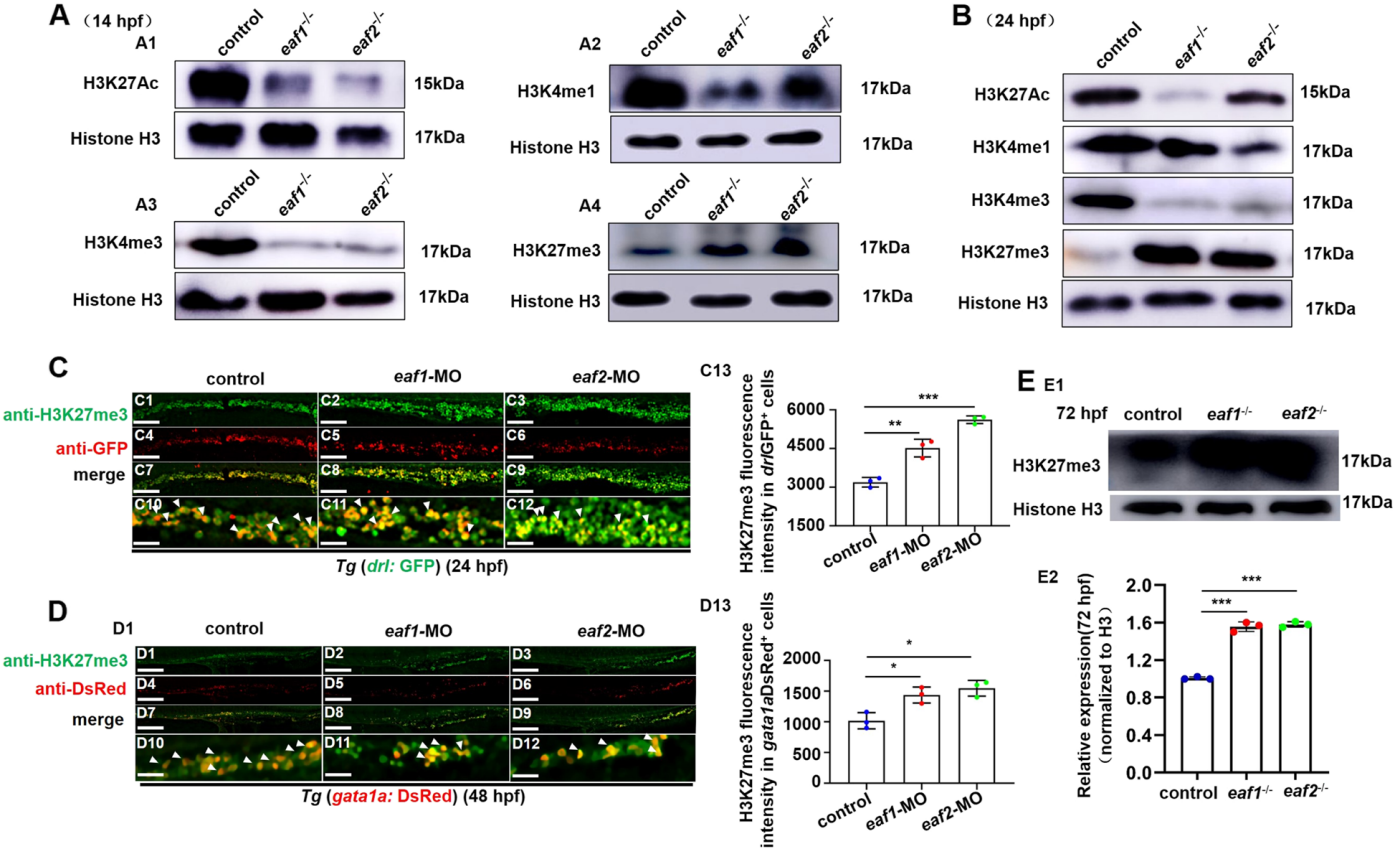
Effects of ***eaf1/2*** deficiency on the protein levels of H3K27ac, H3K4me1, H3K4me3, and H3K27me3.** A** Protein levels of H3K27ac, H3K4me1, H3K4me3, and H3K27me3 in *eaf1*^−/−^, *eaf2*^−/−^, and WT embryos at 14 hpf (A1-A4) and at 24 hpf (**B**). **C**,** D** Double staining of *drl*GFP^+^ and H3K27me3 (C1-C12), and *gata1a*DsRed^+^ and H3K27me3 (D1-D12), in the control and embryos injected with *eaf1*-MO and *eaf2*-MO at 24 hpf or 48 hpf, and quantification of H3K27me3 immunofluorescence intensities in *drl*GFP^+^ cells (C13) and *gata1a*DsRed^+^ cells (D13), with white arrowheads indicating double-positive cells. C10-C12 and D10-D12 show the magnified views of C7-C9 and D7-D9, respectively. **E** Western blotting analysis of H3K27me3 protein level in *eaf1*^−/−^, *eaf2*^−/−^ and WT larvae at 72 hpf (E1), and quantification of H3K27me3 (E2). Each experiment was repeated at least three times, with similar results for two or three replicates, and a representative result are shown. Data are mean ± SD. C1-C12, D1-D12, lateral view, anterior to the left. *P < .05, **P < .01, ***P < .001. NS, not significant. Scale bar = 100 μm (C1-C9, D1-D9) and 50 μm (C10-C12, D10-D12)

**Fig. 7:**
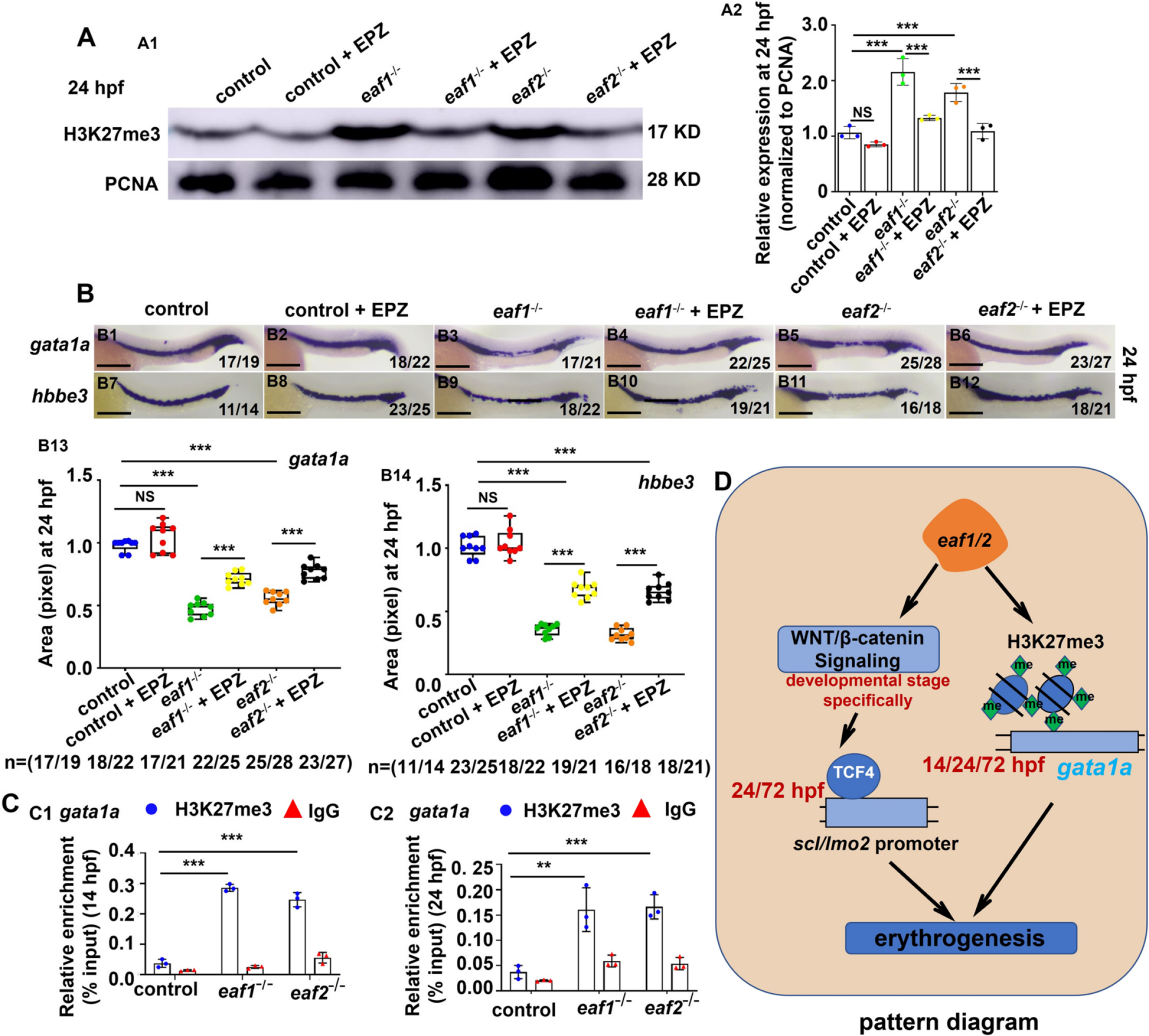
H3K27me3 protein level and expression of ***gata1a*** and ***hbbe3*** could be recovered effectively by H3K27 methylation inhibitor (EPZ005687) in ***eaf1***^−/−^ and ***eaf2***^−/−^ mutants.** A** Western blotting analysis of H3K27me3 protein level in *eaf1*^−/−^, *eaf2*^−/−^ and WT larvae, and the corresponding groups treated with EPZ (EPZ005687) at 24 hpf (A1), and quantification of H3K27me3 protein (A2). **B** WISH analysis of the expression of *gata1a* and *hbbe3* in *eaf1*^−/−^, *eaf2*^−/−^, WT embryos and the corresponding groups treated with EPZ (EPZ005687) at 24 hpf (B1-B12), and statistical analysis of WISH results (B13, B14). **C** ChIP-qPCR analysis of the binding enrichment of protein H3K27me3 on the promoter of gene *gata1a* in *eaf1*^−/−^ and *eaf2*^−/−^ embryonic cells at both 14 hpf (C1) and 24 hpf (C2), with anti- IgG used as a negative control. **D** The working model of *eaf1* and *eaf2* in regulating erythropoiesis. Knockout of *eaf1* or *eaf2* causes the reduced RBCs and the changed H3K27me3 - *gata1a* signaling axis, resulting in changes in the binding enrichment of H3K27me3 on the promoter of gene *gata1a* in zebrafish. Meanwhile, *eaf1* and *eaf2* promote zebrafish erythropoiesis by modulating the canonical WNT/β-catenin signaling pathway in a developmental stage-specific manner. Each experiment was repeated at least three times, with similar results for two or three replicates, and a representative result are shown. Data are mean ± SD. B1-B12, lateral view, anterior to the left. *P < .05, **P < .01, ***P < .001. NS, not significant. Scale bar = 200 μm (B1-B12)

Studies have indicated that *eaf1/2* are part of the super elongation complex (SEC) family in transcriptional control and in epigenetic modification (Cucinotta and Arndt 2016; Luo et al. 2012; Zheng et al. 2021). Thus, we tested the protein levels of epigenetic modified proteins (H3K27Ac, H3K4me1, H3K4me3, and H3K27me3) in *eaf1*^*−/−*^, *eaf2*^−/−^, and control embryos at 14 hpf, 24 hpf and 72 hpf. Compared with the control, *eaf1*^*−/−*^ and *eaf2*^−/−^ embryos showed significant (*P* < .001) reduction in the level of H3K27Ac, H3K4me1, and H3K4me3, in contrast to upregulation (*P* < .05) in the protein level of H3K27me3 at both 14 hpf and 24 hpf (Figs. 6A-B, S10A-B). Meanwhile, knockdown of either *eaf1* or *eaf2* led to decreased number of both *drl*^+^ and *gata1a*^+^ cells accompanied by increased H3K27me3 level in the cells (Figs. 6C-D). Similar results were observed in *eaf1* and *eaf2* mutants or morphants at 72 hpf (Figs. 6E, S10C). In addition, H3K27me3 protein level and expression of *gata1a* and *hbbe3* could be recovered effectively by H3K27 methylation inhibitor (EPZ005687) in *eaf1*^−/−^ and *eaf2*^−/−^ mutants at 24 hpf (Figs. 7A, B).

Whether the increased expression of H3K27me3 could affect the expression of *scl*, *lmo2*, and *gata1a* in *eaf1*^*−/−*^ and *eaf2*^−/−^ mutants was tested by ChIP-qPCR analysis of the binding enrichment of H3K27me3 on *scl*, *lmo2*, or *gata1a* promoter respectively. In Fig. 7C, H3K27me3, a histone marker for transcription silence, was seen to be highly enriched (*P* < .001) in *gata1a* promoter, in contrast to no obvious changes in the enrichment of H3K27me3 in both *scl* and *lmo2* promoters in *eaf1*^*−/−*^ and *eaf2*^−/−^ embryos relative to the WT at both 14 hpf and 24 hpf (Fig. S10D). These results suggested that disrupting zebrafish *eaf1* and *eaf2* may affect the enrichment of H3K27me3, especially on the promoter of *gata1a* rather than the promoters of *scl* and *lmo2*, indicating that *eaf1* and *eaf2* may modulate *gata1a* transcription by modulating H3K27 trimethylation.

We also tested the expressions of erythropoiesis markers and Wnt signaling members in embryos with ectopic expression of *eaf1* or *eaf2* mRNA at primitive and definitive erythropoiesis stages. Overexpression of either *eaf1* or *eaf2* produced opposite effects on the expression of erythropoiesis genes in mutants, such as erythropoiesis transcriptional factors *lmo2, gata1a, scl*, and Wnt indicator *axin2* (Fig. S11), and caused increased Top-GFP expression opposite to those of *eaf1* or *eaf2* morphants (Fig. S12A). Meanwhile, the increased expression of erythrocyte hemoglobin gene *hbbe3* was also observed in embryos with ectopic expression of either *eaf1* or *eaf2* (Fig. S11). However, there were no significant difference for hypoxia tolerance among the WT and groups with ectopic expression of either *eaf1* or *eaf2* (Fig. S12C).

## Discussion

Many previous studies have focused on the role of the *EAF* genes in tumor suppressor and transcriptional properties (Heydaran et al. 2021; Liu et al. 2020), but paid little attention to the linkage of EAF1 and EAF2 dysfunction with erythropoiesis and hypoxia tolerance. In the study, we unveiled that (1) specific erythrogenesis defects occurred in *eaf1*^*−/−*^ and *eaf2*^−/−^ mutants; (2) loss of *eaf1* and *eaf2* in zebrafish reduced their hypoxia tolerance and caused dynamic changes in WNT/β-catenin signaling activities during erythropoiesis; (3) the changed expression of WNT/β-catenin signaling reduced the binding enrichment of WNT/β-catenin transcriptional factor TCF4 on the promoters of *scl* and *lmo2*, meanwhile, the H3K27me3 immunofluorescence intensity in *drl*^+^ and *gata1a*^+^ cells and the binding enrichment of histone H3K27me3 on the promoter of *gata1a* increased, which contributed jointly to the reduced expression of *scl*, *lmo2*, and *gata1a*, leading to defective erythropoiesis and reduced hypoxia tolerance in the mutants.

Erythrocytes play an important role in transporting oxygen and exporting metabolites in vivo (Baron 2013). Impairment in the generation of erythrocytes, a process known as erythropoiesis, or in hemoglobin synthesis, can alter cell function to reduce oxygen supply and lead to low oxygen resistance (Fago 2017). Consistently, depletion of *eaf1* and *eaf2* resulted in a severe reduction in the number of erythrocytes and hypoxia tolerance in zebrafish. This is consistent with a previous report that *eaf1* knockdown disrupts primitive hematopoiesis (Hu et al. 2014). In addition, *eaf1* mRNA could effectively rescue the erythrocytes’ marker *gata1a* and *hbbe3* in both *eaf1*^*−/−*^ and *eaf2*^*−/−*^ mutants, and *vice verse*. Embryos with knockdown of both *eaf1* and *eaf2* exhibited more reduced expression in *gata1a* and *hbbe3* compared with embryos with knockdown of either *eaf1* or *eaf2*. Additionally, the decrease of bata-Catenin fluorescence intensity in *gata1a*^*+*^ cells and of Top-GFP activities in the brain and spinal cord was more substantial in the embryos with knockdown of both *eaf1* and *eaf2*, suggesting *eaf1* and *eaf2* may play redundant roles in erythropoiesis in zebrafish, consistently with the reports in *Arabidopsis thaliana* (Dabas et al. 2021) and in plasma cells (Arumemi et al. 2013) that EAF1 and EAF2 share some redundantly biological roles. Expression of related family genes is upregulated by genetic compensation mechanism when one homology is knockout (El-Brolosy et al. 2019; Ma et al. 2019). In this study, the transcriptional adaptation between *eaf1* and *eaf2* in erythrocyte progenitor *drl*^+^ or *gata1a*^+^ cells was also observed, although we could not observe the transcriptional adaptation between *eaf1* and *eaf2* in the whole embryos.

*Scl* and *Lmo2* are required for erythrocyte development (Dooley et al. 2005; Patterson et al. 2007; Tijssen et al. 2011), and *gata1a* is essential for erythrocyte development in zebrafish (Ferreira et al. 2005). In the present study, significant reduction was observed in the expression of erythrogenesis transcriptional regulator *gata1a* at 14 hpf, 24 hpf, and 72 hpf, as well as the erythrogenesis transcriptional regulators *scl* and *lmo2* at 24 hpf and 72 hpf, and the reduced expression of these three endpoint regulators (*gata1a, scl* and *lmo2*) in erythrogenesis certainly led to reduced primitive and definitive erythrogenesis in *eaf1*^*−/−*^ and *eaf2*^−/−^ mutants. Meanwhile, *eaf1/2* deletion caused reduction in RBCs, coupled with an increased number of hematopoietic stem/progenitor cells and unchanged expression of vascular markers, suggesting that *eaf1/2* deletion might disrupt the differentiation and formation of RBCs, but not influence the commitment of HSPCs and vascular cells, inferring that *eaf1/2* deletion specifically affects erythrogenesis.

Previous studies have shown that zebrafish EAF1 and EAF2 regulate anterior and posterior pattern during zebrafish embryogenesis by suppressing canonical WNT/β-catenin signaling, which might be the mechanism for EAF1 and EAF2 in tumor suppression (Liu et al. 2013). In this study, we found that depletion of *eaf1* or *eaf2* resulted in a notable upregulation of gene *axin2* at 16 hpf while downregulation at 24 hpf and 72 hpf. *Axin2* is a pivotal WNT/β-catenin activity indicator in cells, with an important role in the regulation of β-catenin stability in the WNT/β-catenin pathway. Consistently, we also found obviously increased level of P-β-Catenin-Ser552 at 16 hpf, which is a pivotal indicator of active Wnt signaling (Ahmadzadeh et al. 2016), but its level was almost undetectable in the two mutants at 24 hpf. Consistently, TopFlash activities increased at 16 hpf while significantly reduced at 24 hpf in both *eaf1*^*−/−*^ and *eaf2*^−/−^ mutants. Additionally, β-Catenin level was obviously reduced in RBCs from embryos and larvae with functional deficiency of either *eaf1* or *eaf2* at both 24 hpf and 72 hpf, further suggesting that depletion of *eaf1* or *eaf2* caused dynamic changes in WNT/β-catenin activities during erythrogenesis, with upregulation first at 16 hpf and then downregulation at 24 hpf and 72 hpf, which might jointly contribute to the primitive and definitive erythrogenesis defects in *eaf1*^*−/−*^ and *eaf2*^−/−^ mutants.

In *eaf1*^*−/−*^ and *eaf2*^*−/−*^ mutants, the WNT/β-catenin signaling targets *scl* and *lmo2* were significantly upregulated at 14 hpf, but downregulated at 24 hpf. Additionally, ChIP-qPCR assays revealed that depletion of Eaf1 or Eaf2 caused significant reduction in the binding enrichment of TCF4 on promoters of the genes *scl* and *lmo2*, which might lead to the significantly reduced expression of both *scl* and *lmo2* in the mutants at 24 hpf, contributing to defective erythrogenesis in the mutants.

Previous studies have also shown that epigenetic modifications function importantly in erythropoiesis (Ge et al. 2014; Hu et al. 2009; Wang et al. 2021; Wong et al. 2011; Yang et al. 2019) and EAF1/2 are part of the super elongation complex (SEC) family with epigenetic modification function (Cucinotta and Arndt 2016; Luo et al. 2012; Zheng et al. 2021). In this study, the protein levels of H3K4me1, H3K4me3, H3K27ac, and H3K27me3 are changed in the *eaf1*^−/−^ and *eaf2*^*−/−*^ mutants, with downregulation for H3K4me1, H3K4me3 and H3K27ac while up-regulation for H3K27me3. H3K4me1 is an enhancer marker (Bae & Lesch 2020), H3K4me3 is a promoter marker (Ruthenburg et al. 2007), H3K27ac is a marker of both promoter and enhancer (Creyghton et al. 2010), and H3K27me3 is a marker for gene suppression (Gan et al. 2015; Zhang et al. 2022). In this study, we observe increased level of H3K27me3 protein in erythrocyte progenitor *drl*^+^ and *gata1a*^+^ cells at both 24 hpf and 72 hpf, and find the binding enrichment of H3K27me3 on *gata1a* promoter is significantly increased at both 14 hpf and 24 hpf, in contrast to no obvious change in the binding enrichment of H3K27me3 on *scl* or *lmo2* promoters in *eaf1*^−/−^ and *eaf2*^*−/−*^ embryonic cells, indicating that *eaf1* and *eaf2* could activate *gata1a* rather than *scl* or *lmo2* transcription by suppressing the epigenetic modified protein H3K27me3 during primitive and definitive erythrogenesis. Meanwhile, we also observe the impaired response of hypoxia-inducible genes *hif1αb*, *hif2αb*, *hif3α*, *cited2*, *pai1* and *ldha* in the mutants *eaf1*^−/−^ and *eaf2*^*−/−*^ after hypoxia stimulation, which not only might be another contributor for the reduced hypoxia tolerance occurred in the mutants, but also consistent with previous reports that *EAF* family genes are required for the normal expressions of hypoxia-inducible genes (Chen et al. 2014).

This study provides impartial evidence that Eaf1 and Eaf2 regulate zebrafish erythrogenesis by modulating the expression of *scl* or *lmo2* and WNT/β-catenin signaling in a developmental-stage-specific manner. However, why WNT/β-catenin signaling activity is upregulated at 16 hpf but downregulated from 24 hpf during erythrogenesis process is still unknown and needs to be further elucidated in future studies. In this study, we unveil a function of *eaf1* and *eaf2* in erythropoiesis and hypoxia tolerance in zebrafish. Our study provides new insights into the molecular mechanism underlying erythropoiesis, which not only has general implications in regeneration medicine of anemia and related diseases, but also provides evidence that genes *eaf1* and *eaf2* are important molecules in modulating fish economic or productive traits, such as growth, disease resistance, hypoxia tolerance, and so on (Gui et al. 2021; Kafina and Paw 2018; Patton et al. 2021; Zhang et al. 2021).

## Conclusions

In summary, we found that Eaf1 or Eaf2 dysfunction caused reduced RBCs and hypoxia tolerance in zebrafish. Loss of *eaf1* and *eaf2* caused significant changes in the expression of epigenetic modified histones, with a significant increase of H3K27me3 enrichment in *gata1a* promoter. Meanwhile, deficiency of *eaf1* or *eaf2* resulted in a dynamic expression of canonical WNT/β-catenin signaling during erythropoiesis, with reduced β-Catenin level and enrichment of the WNT transcriptional factor TCF4 in both *scl* and *lmo2* promoters.This study not only has general implications in regeneration medicine of anemia and related diseases, but also provides evidence that genes *eaf1* and *eaf2* are important molecules in modulating fish economic or productive traits, such as growth, disease resistance, hypoxia tolerance, and so on.

## Methods

The full names and abbreviations of genes tested in this study are listed in Table S1.

### Zebrafish strains

The ages of embryos and larvae were expressed by hours post-fertilization (hpf), days post-fertilization (dpf), and months post-fertilization (mpf).

### Generation of *eaf2* mutant zebrafish embryos by CRISPR/Cas9 system

In this study, *eaf1*^*−/−*^ (Δ1, -5) was used as we described previously (Liu et al. 2018), and *eaf2*^*−/−*^ (Δ2, -10) mutants were generated using the CRISPR/Cas9 system using the following guide RNA (gRNA) targeting sequence: 5’- CGGGAGGAGAGCTCTTGGTGCTGGA − 3’, with the gRNA synthesized using T7 RNA polymerase. The mixture of gRNA target (500 ng/µL) and Cas9 protein (600 ng/µL) was co-injected into one-cell stage embryos, followed by raising the injected embryos to sexual maturity and screening for a stable F2 homozygous line. Genotyping assays of *eaf2* heterozygote and homozygous mutants were performed using the primers listed in Table S2. Wild-type (WT) (AB line), *eaf1*^*−/−*^ and *eaf2*^−/−^zebrafish were maintained under standard conditions as described previously (Liu et al. 2009). Male and female zebrafish were kept separately until mating and spawning. Embryos were obtained by natural spawning and cultured at 28.5℃ in an incubator.

### Drug treatment

In this study, Bio (6-Bromoindirubin-3’-oxime) (B1686, Sigma-Aldrich) was prepared as described previously (Liu et al. 2013; Zhang et al. 2020). EPZ005687 (E125682, Aladdin) was dissolved in DMSO (D2650, Biosharp). Embryos from the control, *eaf1*^−/−^ and *eaf2*^−/−^ mutants at bud stage were exposed to BIO (0.05 µM) and EPZ005687 (2 µM) respectively, and were harvested at indicated stages. Biological replicates were performed 3 times with over 10 embryos per group one time.

### Quantitative real-time PCR

To determine the expression of *eaf1* and *eaf*2 in *eaf1*^*−/−*^, *eaf2*^−/−^ and WT embryos, expression of *hif1αb*, *hif2αb*, *hif3α*, *cited2*, *pai1* and *ldha* in *eaf1*^*−/−*^, *eaf2*^−/−^ and WT embryos or larvae under hypoxia, and expression of *gata1a*, *hbbe3*, *lmo2*, *scl*, *axin2*, *fzd3a* and *znf703* in the embryos injected with *eaf1* mRNA or *eaf2* mRNA at 14 hpf and 24 hpf, and qRT–PCR was conducted as we reported previously (Liu et al. 2017; Zhang et al. 2020). The primer sequences are listed in Table S3, and the primer sequences of *cited2*, *pai1* and *ldha* have been reported previously (Cai et al. 2018 ). Each sample was run in triplicate and repeated at least three times. Differences were calculated by the ΔΔCt comparative quantization method using β-actin as an internal control.

### One step cell-direct qRT–PCR

In this study, *Tg* (*drl*: GFP) and *Tg* (*gata1a*: DsRed) embryos at 24 hpf or 48 hpf were disaggregated into suspended single cells using our previously reported method (Chen et al. 2019). The GFP-positive cell (*drl*^+^ cells) and DsRed-positive cells (*gata1a*^+^ cells) (5000–10,000 sorted cells/sample) were sorted into the lysis solution provided by the CellsDirect™ One-Step qRT–PCR Kit (Invitrogen, 11753-100) using fluorescence-activated cell sorting based flow cytometry (FACS) (BD FacsAria SORP, 650110M3, BioDot, USA). The lysates were used as template for one step cell-direct qRT–PCR. Primer sequences of the tested genes are shown in Table S3, including *eaf1*, *eaf2*, *gata1a*, *hbbe3*, *tcf4*, *myod* and *olig2*. One step cell-direct qRT–PCR was performed as we reported previously (Chen et al. 2019).

### Hypoxia treatment

In this study, the hypoxia treatment followed a previously reported method (Cai et al. 2020). Briefly, the InvivO2 300 Hypoxia Workstation was used for hypoxia treatment of zebrafish embryos and larvae (24 hpf and 72 hpf) and adults (6 mpf), with the O_2_ concentration adjusted to the appropriate value (2% for larvae and 5% for adults) before the experiments. The adult zebrafish (6 mpf) with a similar body weight (0.30 ± 0.02 g) were selected for the hypoxia tolerance tests, where *eaf1*^*−/−*^, *eaf2*^−/−^, and WT adult zebrafish were placed separately into 250 mL flasks, each containing 250 mL of water, with 3 adult zebrafish per flask.

For embryos and larvae hypoxia tolerance test, *eaf1*^*−/−*^, *eaf2*^−/−^, and WT zebrafish larvae were placed into a 60 mm cell culture dish filled with 5 mL of water, with 30 larvae per dish. Before the experiment, the oxygen concentration in the InvivO2 300 Hypoxia Workstation was adjusted to 2%, and each experiment was repeated three times. Meanwhile, *eaf1*^*−/−*^, *eaf2*^−/−^, and WT larvae or adult zebrafish exposed to normoxia (21% O_2_) were used for comparison.

### Oxygen consumption of adult zebrafish

We measured the zebrafish oxygen consumption of WT, *eaf1*^*−/−*^, and *eaf2*^−/−^ mutants in 250 mL flasks (each containing 250 mL of water). The initial oxygen concentration in the water of each flask was measured with an LDO101 probe (HQ40d, HACH) (8.00 ± 0.12 mg/L). For this experiment, a total of 18 adult zebrafish (6 *eaf1*^*−/−*^; 6 *eaf*^*−/−*^, and 6 WT siblings) with a similar body weight were selected and placed separately in 18 flasks, followed by sealing the flasks tightly. After 4 h (h), we measured the oxygen concentration in the 9 flasks containing *eaf1*-null (*eaf1*^*−/−*^), *eaf2*-null (*eaf2*^*−/−*^) and WT zebrafish siblings (1 zebrafish per flask) with the LDO101 probe. After 8 h, we measured the oxygen concentration individually in the remaining nine flasks with the LDO101 probe.

### Flow cytometry

In this study, adult WKM samples of *eaf1*^−/−^, *eaf2*^−/−^ and WT zebrafish were prepared as described (Hou et al. 2017; Traver et al. 2003). Briefly, cell suspensions of WKM were obtained by aspiration using a 1-mL syringe in ice-cold 1×PBS containing 5% FBS, and then filtered using a 40-µm mesh. Samples were stained with propidium iodide (Invitrogen, USA) to exclude dead cells and debris, and were analyzed using a CytoFLEX Flow Cytometer (Beckman Coulter, USA).

### O-dianisidine staining

To detect the hemoglobin level in living embryos, o-dianisidine staining (D9143, Sigma-Aldrich) was used to indicate the hemoglobin in the *eaf1*^*−/−*^, *eaf2*^*−/−*^ and control embryos at 36, 48, 60, 72, and 96 hpf as previously reported (Amatruda and Zon 1999; O’brien 1961; Zhou et al. 2016). After staining, a rust-colored precipitate (labeled hemoglobin) appeared specifically in erythroid cells, and the treated embryos with a lighter colored precipitate were defined as embryos with reduced erythrocytes (hemoglobin). Next, the embryos were transferred to 100% glycerol for stereoscopic observation and photographing, followed by calculating the percentage of embryos with reduced hemoglobin as reported previously (Zhang et al. 2015).

### Morpholino (MO) and mRNA synthesis

The *eaf1* and *eaf2* MO sequences have been reported previously (Liu et al. 2013). The full-length *eaf1* and *eaf2* were amplified with the specific primers shown in Table S4, and synthesized using the Ambion MAXIscript T7 Kit (Cat#AM1344, Invitrogen, USA) as instructed by the manufacturer. The MOs and mRNAs were injected into one-cell stage embryos, with the MO dose of *eaf1* or *eaf2* at 8 ng/embryo, and the mRNA concentration of *eaf1* or *eaf2* at 200 pg/embryo as we reported previously (Liu et al. 2013).

### Whole-mount *in situ* hybridization (WISH)

WISH detection followed our previously reported method (Jin et al. 2021) using our recently reported genes as probes: *hbbe1* (hemoglobin beta embryonic-1.1), *hbbe2* (hemoglobin beta embryonic-2), *hbbe3* (hemoglobin beta embryonic-3), *scl* (T-cell acute lymphocytic leukemia 1), *lmo2* (LIM domain only 2), *gata1a* (GATA binding protein 1a), *gata2* (GATA binding protein 2a), *fli1* (Fli-1 proto-oncogene), *flk1* (kinase insert domain receptor like), *c-myb* (v-myb avian myeloblastosis viral oncogene homolog), *runx1* (RUNX family transcription factor 1), *rag1* (recombination activating 1), *myod* (myogenic differentiation 1), *wnt3* (wingless-type MMTV integration site family, member 3), *wnt16* (wingless-type MMTV integration site family, member 16), *fzd2* (frizzled class receptor 2), *axin2* (conductin, axil), and so on (Galloway et al. 2005; Jin et al. 2021; Zhang et al. 2018; Zhou et al. 2016). Some probes were amplified from the cDNA pool using the primers displayed in Table S5. For WISH data, the number in the right-down corner in each panel in WISH figures in the experimental groups was shown as N_changed_/N_total_, where N_changed_ indicates the number of embryos with reduced or increased expression, and N_total_ indicates the total number of embryos in a group; the number in WISH figures in the control groups was shown as N_normal_/N_total_, where N_normal_ indicates the number of embryos with normal expression and N_total_ indicates the total number of embryos in a group.

### Western blot

Embryos at 14 hpf and 24 hpf were homogenized using RIPA (Radio Immunoprecipitation Assay) lysis buffer (10 mM Tris–HCl, 10% glycerol, 1% SDS, 1% Chap; G3423, GBCBIO, China) with proteinase inhibitor (P2714, Roche), followed by adding appropriate SDS-PAGE loading buffer, boiling the obtained protein for 10 min, and separating an almost equal amount of protein in each line by polyacrylamide gel electrophoresis. After transferring the separated protein to a polyvinylidene fluoride microporous membrane (Bio-Rad Laboratories, Hercules, CA, USA), the blots were blocked with 0.5% skim milk in TBS containing 0.1% Triton X-100, followed by incubation first with the primary antibodies (1:1000), and then with secondary antibodies (BL033A, Biosharp 1:1000). Finally, the blots were visualized using enhanced chemiluminescence (Bio-Rad Laboratories, Hercules, CA, USA). The following antibodies were used in the assays: EAF1 (A17798, ABclonal), EAF2 (ab151692, Abcam), HIF-1a (A7553, ABclonal), β-Catenin (AF8340, Affinity), Phospho-β-Catenin-S552 (AP0979, ABclonal), Actin (AC026, ABclonal), GAPDH (AC001, ABclonal) H3K4me1 (A2355, ABclonal), H3K4me3 (A2357, ABclonal), H3K27me3 (A2363, ABclonal), H3K27ac (A7253, ABclonal), anti-H3 (A2348, ABclonal).

### Luciferase reporter assays

Luciferase reporter assays were performed as described previously (Liu et al. 2018; Liu et al. 2013). 8xTopFlash reporter (25ng/uL) and pTK-renilla (5ng/uL) were co-injected into one-cell stage embryos as we performed previously (Liu et al. 2013). The luciferase activities of 8xTopFlash reporter in *eaf1*^*−/*−^, *eaf2*^−/−^, and WT embryos at 16 hpf and 24 hpf were measured using the Dual-luciferase Reporter Assay System (DL101, Vazyme) following the protocol of the manufacturer. The data were reported as the mean ± SD of three independent experiments in triplicate (Liu et al. 2018).

### Immunofluorescence

Immunofluorescence of whole-mount zebrafish embryos followed a previously reported method (He et al. 2020). In this study, *Tg* (*drl*: GFP) embryos at 24 hpf and *Tg* (*gata1a*: DsRed) embryos at 24/48/72 hpf were collected and fixed in 4% paraformaldehyde overnight, and permeabilized with 1 mg/mL collagenase (AC15L141, Life- iLab Biotech, China) for 25 min and blocking in 3% BSA for 1 h. Then the embryos were incubated with anti-GFP (AE011, ABclonal)/anti-DsRed (AE002, ABclonal) and anti-H3K27me3 (A2363, ABclonal)/beta-Catenin (8480, Cell Signaling Technology) overnight at 4 ℃, respectively. After washing with PBST, the embryos were incubated with Alexa Fluor 555-conjugated anti-mouse (AS057, ABclonal) and FITC-conjugated anti-rabbit antibodies (BL033A, Biosharp, China). Images were captured using a Leica TCS SP8 confocal laser microscope (Wetzlar, Germany).

### ChIP-qPCR

Chromatin immunoprecipitation-qPCR **(**ChIP-qPCR) assays were performed as we reported recently (Jin et al. 2021). The chorions of ~ 500 14/24 hpf *eaf1*^*−/−*^, *eaf2*^−/−^, and WT embryos were removed separately by pronase, followed by collecting the cross-linked cells from the dechorinated eggs, washing the cells twice with 1xPBS, obtaining the precipitation by centrifugation, and successive treatment for 10 min separately in lysis buffer 1 (50 mM HEPES-KOH pH 7.5, 140 mM NaCl, 1 mM EDTA, 10% glycerol, 0.5% NP-40, 0.25% Triton X-100) and lysis buffer 2 (10 mM Tris-HCl pH 8.0, 200 mM NaCl, 1 mM EDTA, 0.5 mM EGTA). Next, the pellet was suspended in 1 mL nucleus lysis buffer 3 (10 mM Tris-HCl pH 8, 100 mM NaCl, 1 mM EDTA, 0.5 mM EGTA, 0.1% Na-Deoxycholate, 0.5% N-lauroylsarcosine), followed by sonication to obtain ~ 200–500 bp chromatin DNA fragments.

After sonication, the input control, TCF4 (A1141, China, ABclonal Technology), H3K27me3 antibody (A2363, China, ABclonal Technology), and IgG (Beyotime Inc, China) ChIP groups were treated as described previously (Jin et al. 2021). Finally, the ChIP DNA was recovered by phenol/chloroform/isoamylal-cohol (25:24:1) extraction and precipitated by ethanol. The pellet was re-suspended in water and used as a template for qPCR. The tested genes and their primers used for ChIP-qPCR are listed in Table S6, and qPCR and data analysis followed a recently reported method (Jin et al. 2021; Liu et al. 2017).

### Statistical analysis

GraphPad Prism 8.0 and SPSS 20.0 software were used for statistical analysis of the data, such as WISH, immunofluorescence, RT-qPCR and ChIP–qPCR. The significance of changes was estimated by one-way analysis of variance (ANOVA) and multiple sample repeated comparisons. The statistical significance between groups was determined at *P* < .05 (*), *P* < .01 (**) or *P* < .001 (***).

## Supplementary Information

**Additional file 1:** **Fig. S1.** Effects of *eaf1/2* deficiency on the phenotype of zebrafish during embryogenesis and at adult stage. **Fig. S2.** Hypoxia treatment of eaf1^-/-^, eaf2^-/-^, and WT larvae and adults. **Fig. S3.** Effects of *eaf1/2* deficiency on the expressions of hypoxia inducible factor/genes in zebrafish embryos and larvae under hypoxia. **Fig. S4.** Effects of *eaf1/2* deficiency on erythrogenesis in zebrafish. **Fig. S5.** The functional redundancy between *eaf1* and *eaf2* during zebrafish erythropoiesis development. **Fig. S6.** Effects of *eaf1/2* deficiency on the expression of genes *gata2/fli1/flk1/myod*. **Fig. S7.** Effects of *eaf1/2* deficiency on the expression of *runx1*, *c-myb*, *rag1*, *gata1a* and *lmo2*. **Fig. S8.** Effects of *eaf1/2* deficiency on WNT/β-catenin signaling during fish embryogenesis. **Fig. S9.** Immunofluorescence of β-Catenin protein in RBCs (*gata1a*^+^ cells). **Fig. S10.** Effects of *eaf1/2* deficiency on the protein levels of H3K27ac, H3K4me1, H3K4me3, and H3K27me3. **Fig. S11.** Effects of overexpression of *eaf1*, *eaf2* and overexpression of both genes on erythrogenesis and Wnt signaling. **Fig. S12.** Effects of overexpression of *eaf1*, *eaf2* and overexpression of both genes on expression of *gata1a*, *lmo2*, *axin2*, *wnt16* and *fzd2*, and the hypoxic tolerance of the larvae with ectopic expression. **Table S1.** Genes tested in this study. **Table S2.** Sequences of primers for mutated target loci detection. **Table S3.** Sequences of primers for RT-qPCR and One Step Cell-Direct qRT–PCR. **Table S4.** Sequences of primers for full-length CDS. **Table S5.** Primer pairs for WNT Signaling genes examined in the study. **Table S6.** sequences of primer used for ChIP-qPCR.

**Additional file 2**: Effects of *EAF1/2* deficiency on hypoxia tolerance in zebrafish. **Movie S1**. Wild-type (left, WT), *eaf1*^*-/-*^ (middle) and *eaf2*^*-/-*^ (right) zebrafish (3 mpf, body-weight =0.31 ± 0.04g, mean ± SD) showed no obvious difference in behavior during initial hypoxia stress in a hypoxia workstation (5% O_2_), related to Fig. S2B. **Movie S2**. The *eaf1*^*-/-*^ (middle) and *eaf2*^*-/-*^ (right) zebrafish (3 mpf, body-weight =0.31 ± 0.04g; mean ± SD) were dead or dying compared with the active WT zebrafish (left) under hypoxia (5% O_2_) for 50 min, related to Fig. S2B.

## Data Availability

All data generated or analyzed during this study are included in this article.

## Abbreviations

ChIP-qPCR: Chromatin immunoprecipitation-qPCR
RIPA: Radio-Immunoprecipitation Assay
TBS: Tris Buffered Saline
PBS: Phosphate Buffered Saline
WISH: Whole-mount *in situ* hybridization
EGTA: Ethylene Glycol Tetraacetic Acid
BSA: Bovine serum albumin
FACS: Fluorescence-activated cell sorting based flow cytometry
HSPC: Hematopoietic stem and progenitor cell
dpf: days post fertilization
hpf: hours post fertilization
mpf: months post-fertilization
WT: Wild type
RBCs: Red blood cells
Hb: Hemoglobin
TGF-β: Transforming growth factor bate
*drl*: draculin
