# Supplementary material for: Zebrafish ELL-associated factors Eaf1/2 modulate erythropoiesis via regulating gata1a expression and WNT signaling to facilitate hypoxia tolerance
Source: Cell Regen. 2023 Apr 1;12:10. doi: 10.1186/s13619-022-00154-3 (PMC10066051; doi:10.1186/s13619-022-00154-3)
Supplement: Supplementary file 1 — Additional file 1: Fig. S1. Effects of eaf1/2 deficiency on the phenotype of zebrafish during embryogenesis and at adult stage. Fig. S2. Hypoxia treatment of eaf1-/-, eaf2-/-, and WT larvae and adults. Fig. S3. Effects of eaf1/2 deficiency on the expressions of hypoxia inducible factor/genes in zebrafish embryos and larvae under hypoxia. Fig. S4. Effects of eaf1/2 deficiency on erythrogenesis in zebrafish. Fig. S5. The functional redundancy between eaf1 and eaf2 during zebrafish erythropoiesis development. Fig. S6. Effects of eaf1/2 deficiency on the expression of genes gata2/fli1/flk1/myod. Fig. S7. Effects of eaf1/2 deficiency on the expression of runx1, c-myb, rag1, gata1a and lmo2. Fig. S8. Effects of eaf1/2 deficiency on WNT/β-catenin signaling during fish embryogenesis. Fig. S9. Immunofluorescence of β-Catenin protein in RBCs (gata1a+ cells). Fig. S10. Effects of eaf1/2 deficiency on the protein levels of H3K27ac, H3K4me1, H3K4me3, and H3K27me3. Fig. S11. Effects of overexpression of eaf1, eaf2 and overexpression of both genes on erythrogenesis and Wnt signaling. Fig. S12. Effects of overexpression of eaf1, eaf2 and overexpression of both genes on expression of gata1a, lmo2, axin2, wnt16 and fzd2, and the hypoxic tolerance of the larvae with ectopic expression. Table S1. Genes tested in this study. Table S2. Sequences of primers for mutated target loci detection. Table S3. Sequences of primers for RT-qPCR and One Step Cell-Direct qRT–PCR. Table S4. Sequences of primers for full-length CDS. Table S5. Primer pairs for WNT Signaling genes examined in the study. Table S6. sequences of primer used for ChIP-qPCR. [file 13619_2022_154_MOESM1_ESM.docx]

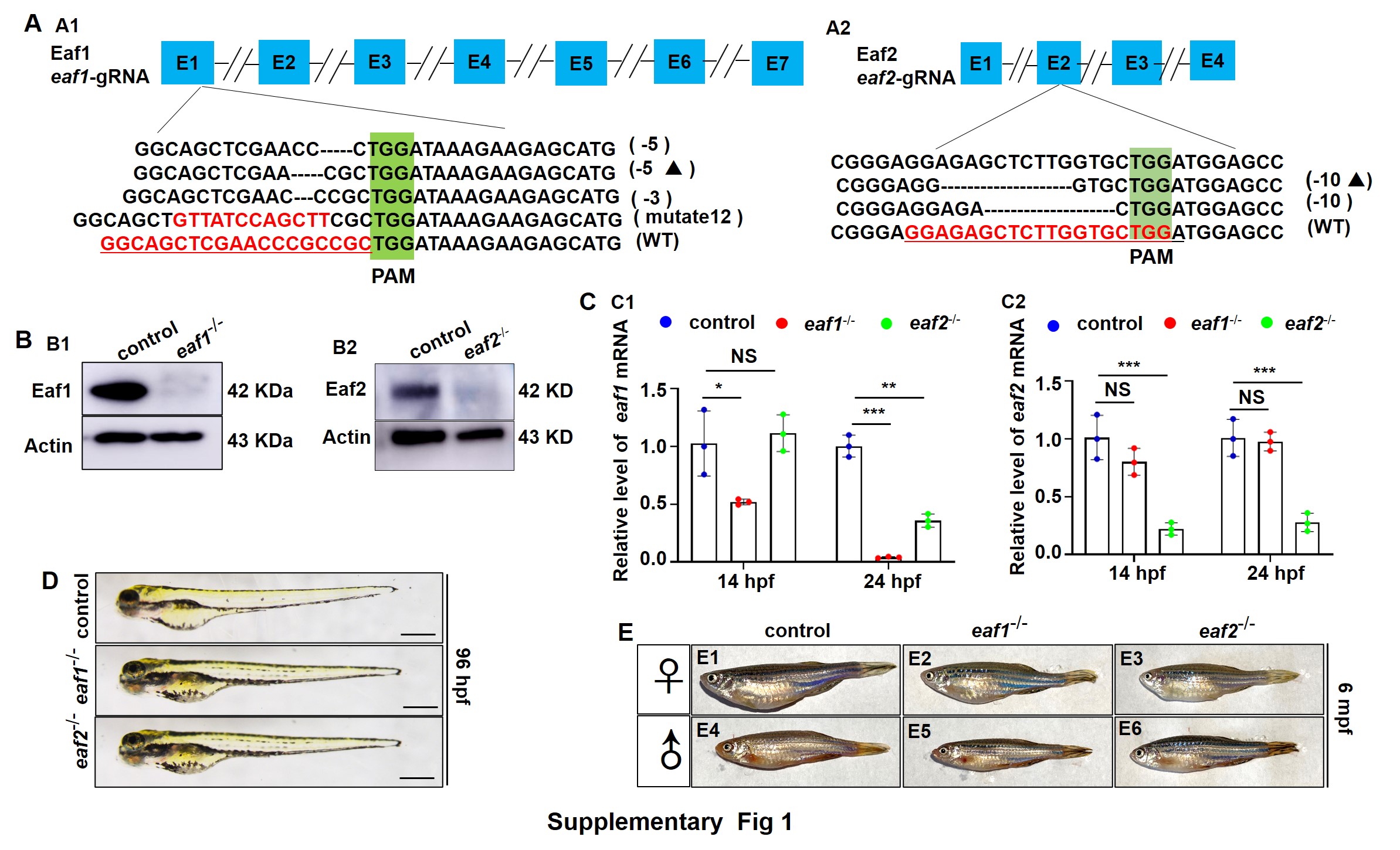


**Fig. S1 Effects of *eaf1/2* deficiency on the** **phenotype of zebrafish during embryogenesis and at adult stage. (A)** The genome structure of mutant *eaf1^-/-^***(A1)** and *eaf2^-/-^* **(A2)**. The triangle symbol marked the genetic mutants for *eaf1^-/-^* and *eaf2^-/-^* used in this study. **(B)** Protein levels of Eaf1 **(B1)** and Eaf2 **(B2)** in *eaf1*^-/-^ and *eaf1*^-/-^ mutants, respectively. **(C)** The transcriptional expression of *eaf1* and *eaf2* in *eaf1^-/-^,* *eaf2^-/-^*, and WT embryos at 14 hpf and 24 hpf, respectively. **(D)** Phenotype of *eaf1^-/-^, eaf2^-/-^*, and WT embryos at 96 hpf. **(E)** Phenotype of *eaf1^-/-^,* *eaf2^-/-^*, and WT zebrafish at 3 mpf. hpf, hours post fertilization; mpf, months post fertilization. Each experiment was repeated at least three times, with similar results for two or three replicates, and a representative result was shown. Data are mean ± SD. **P* < .05, ***P* < .01, ****P* < .001. NS, not significant.


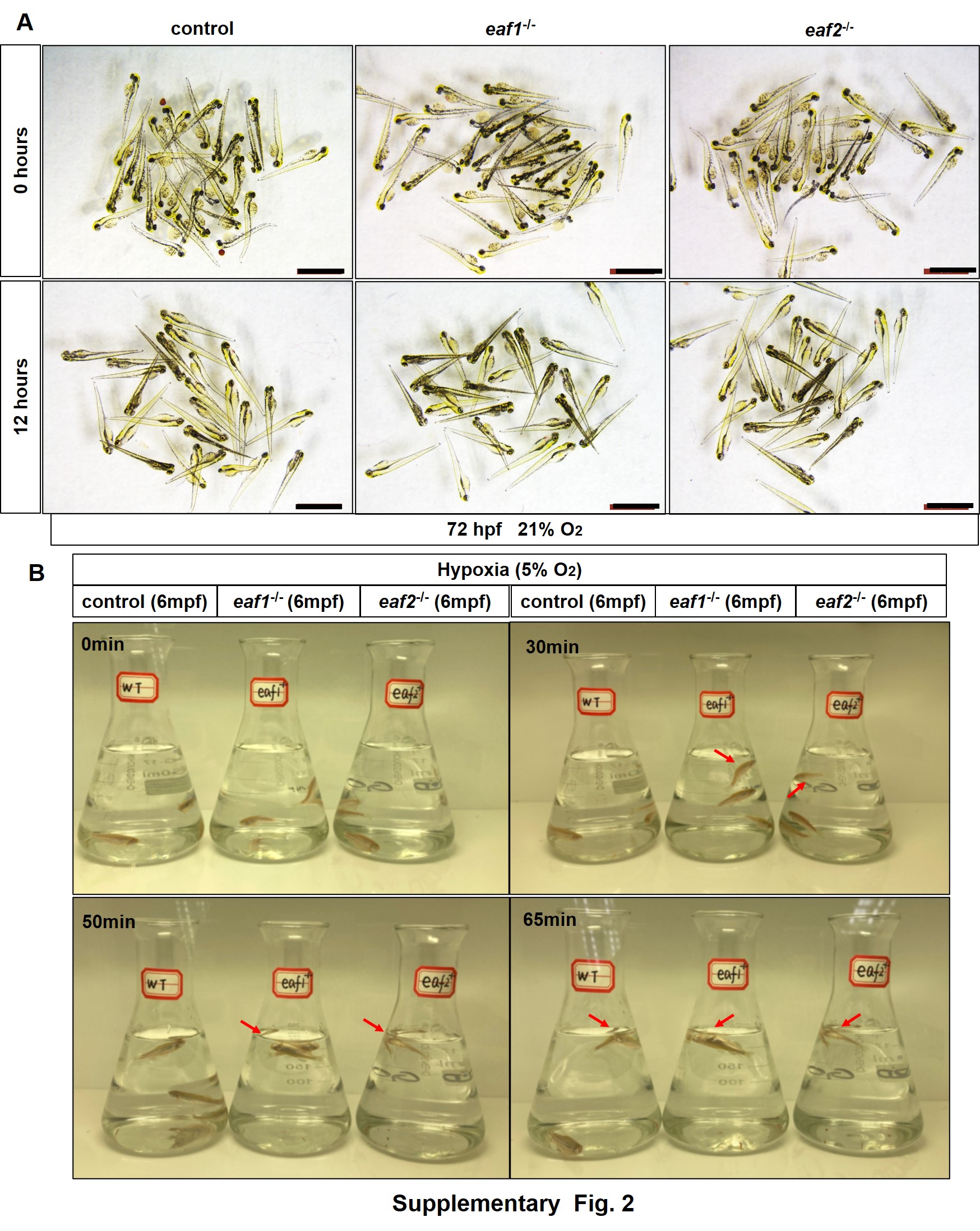


**Fig. S2 Hypoxia treatment of *eaf1*^-/-^, *eaf2*^-/-^, and WT larvae and adults. (A)** Representative images of *eaf1*^-/-^, *eaf2*^-/-^, and WT larvae (30 larvae per group with three replicates, a total of 270 larvae; 3 dpf) exposed to normoxia (21% O_2_) beginning at 72 hpf for 12 h. **(B)** *eaf1*^-/-^and *eaf2*^-/-^adults were more sensitive to hypoxia (5% O_2_) than their WT siblings. Survival of WT and *eaf1*^-/-^ and *eaf2*^-/-^ (6 mpf) after 0 min, 30 min, 50 min, and 65 min in hypoxic conditions (5% O_2_) (three zebrafish per group with three replicates). Red arrows indicate dead zebrafish. Each experiment was repeated at least three times, with similar results for two or three replicates, and a representative result was shown.


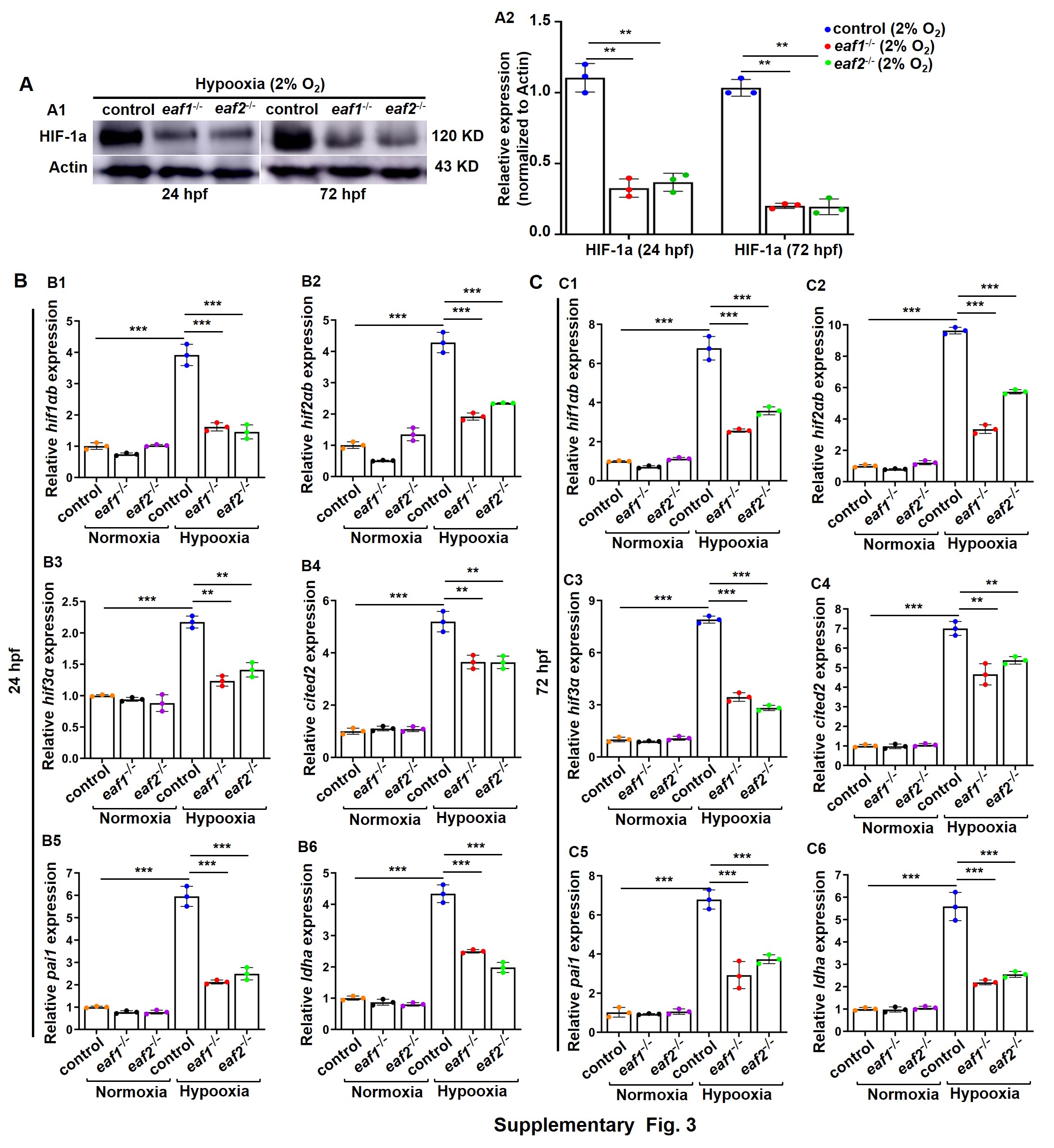


**Fig. S3** **Effects of eaf1/2 deficiency on the** **expressions of hypoxia inducible factor/genes** **in zebrafish** **embryos and larvae under hypoxia. (A)** Protein levels of HIF-1a in *eaf1*^-/-^, *eaf2*^-/-^, and WT embryos (24 hpf) and larvae (72 hpf) under hypoxia (2% O_2_) (**A1**), respectively, and quantitative analysis of protein level in each sample (**A2**). **(B, C)** qRT–PCR analysis of *hif1αb*, *hif2αb*, *hif3α*, *cited2*, *pai1* and *ldha* in *eaf1*^-/-^, *eaf2*^-/-^ and WT embryos **(B1-B6**) and larvae **(C1-C6)** under hypoxia (2% O_2_). Data are presented as mean ± SD. **P* < 0.05, ***P* < 0.01, ****P* < 0.001, NS, not significant.


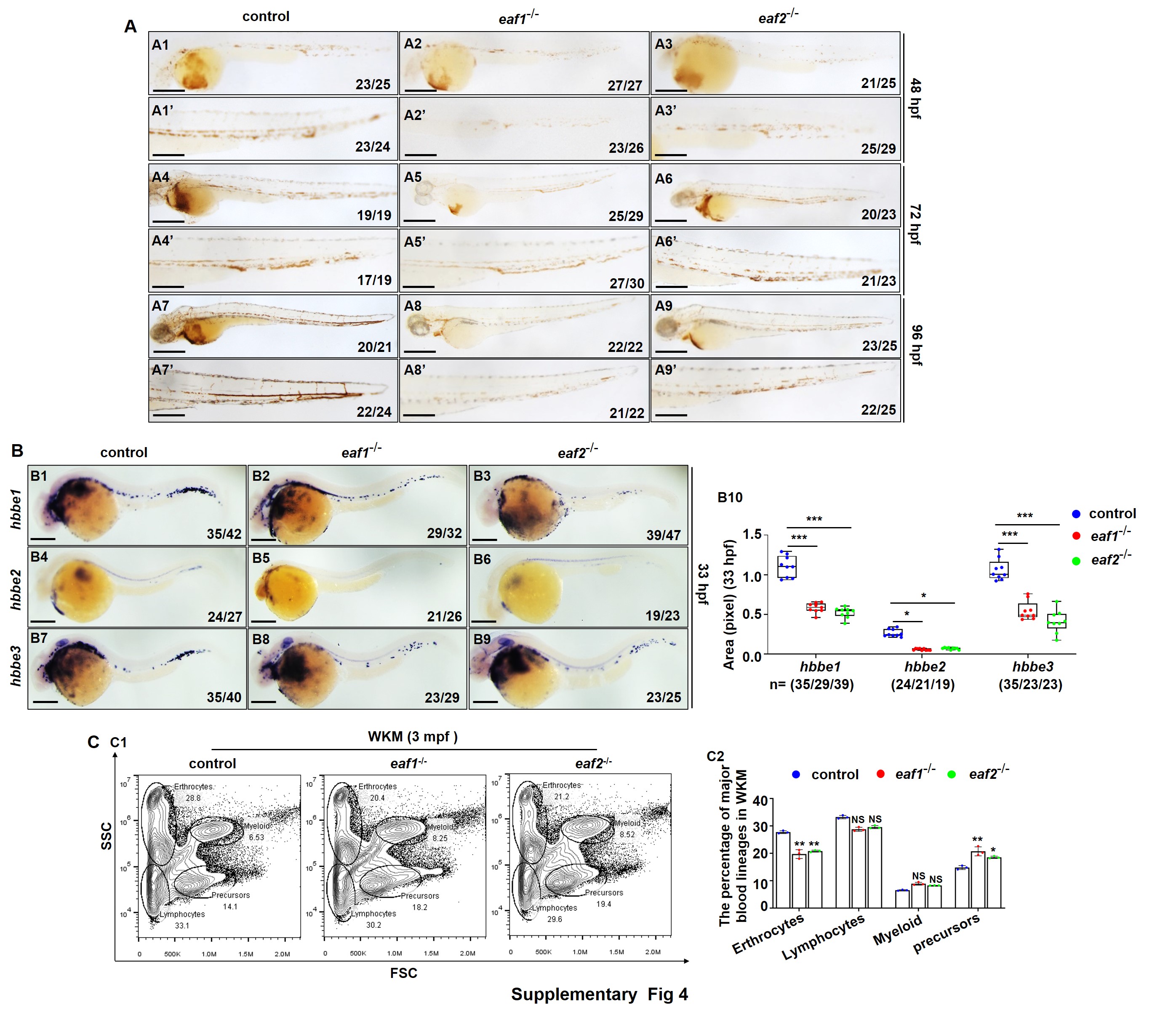


**Fig. S4** **Effects of *eaf1/2* deficiency on** **erythrogenesis in zebrafish. (A)** O-dianisidine staining analysis of erythrogenesis in both *eaf1*^-/-^ and *eaf2*^-/-^ embryos at 48, 72, and 96 hpf relative to WT. **(B)** WISH analysis of the expression of embryonic hemoglobin, *hbbe1/hbbe2/hbbe3* in *eaf1*^-/-^, *eaf2*^-/-^, and WT embryos at 33 hpf **(B1-B9)**, and the statistical analysis of WISH hemoglobin gene staining results **(B10)**. **(C)** Flow cytometry analysis of hematopoietic cells from the adult whole kidney marrow (WKM) in WT, *eaf1*^-/-^ and *eaf2*^-/-^ zebrafish at 3 mpf **(C1),** and percentage of major blood lineages in WKM **(C2)**. Gate populations are as follows: erythrocytes, lymphocytes, myeloid and precursors. Numbers in plots indicate percent of cells in circled gate. Each experiment was repeated at least three times, with similar results for two or three replicates, and a representative result was shown. All embryos are shown in lateral view, anterior to the left, and dorsal to the up. Data are presented as mean ± SD. **P* < 0.05, ***P* < 0.01, ****P* < 0.001, NS, not significant. Scale bar= 200 μm (overall **A1-A9, B1-B9**) and 250 μm (tail **A1’-A9’**).


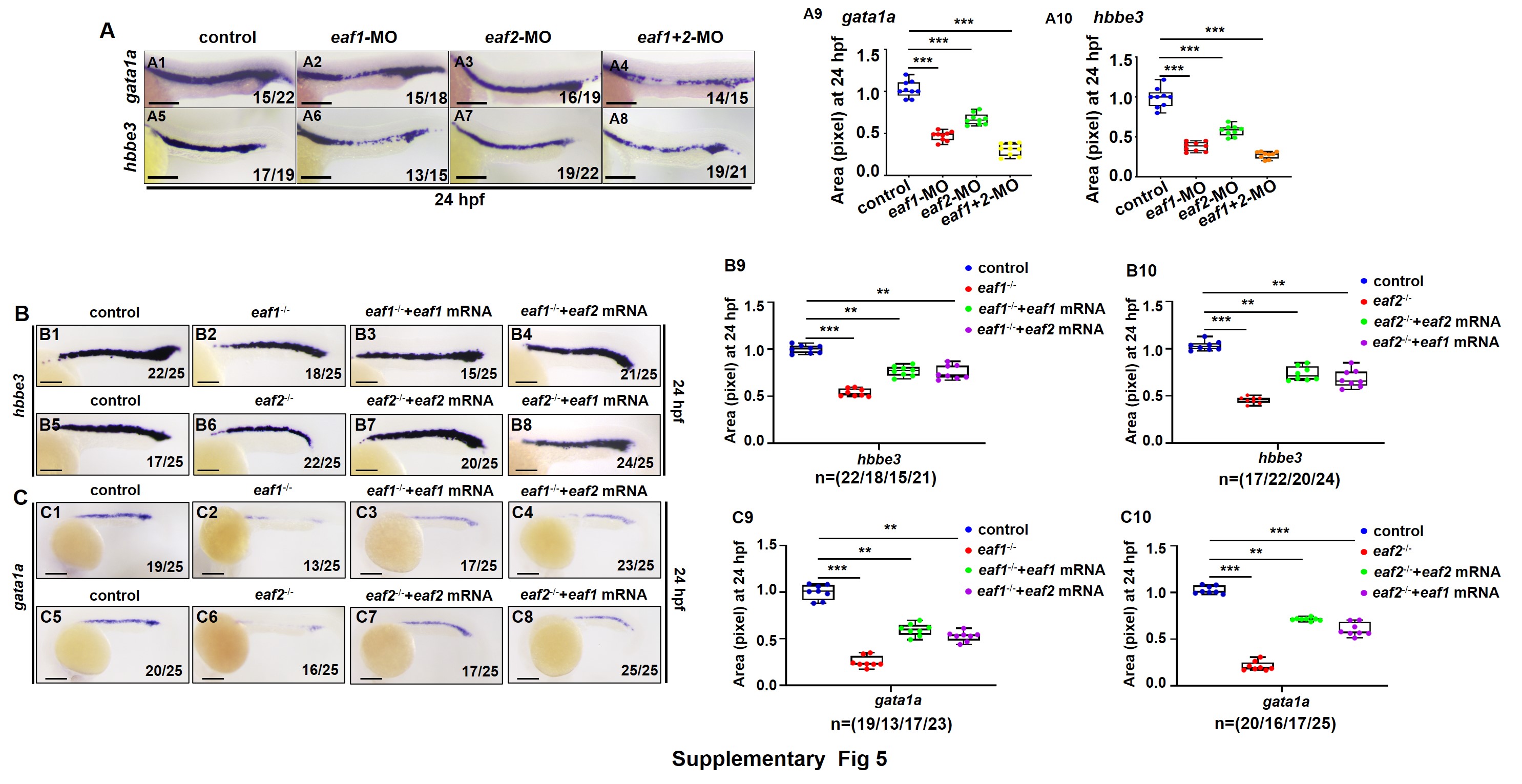


**Fig. S5 The functional redundancy between *eaf1* and *eaf2* during zebrafish erythropoiesis development. (A)** WISH analysis of *gata1a* (**A1**-**A4**) and *hbbe3* (**A5**-**A8**) in the control and embryos injected with *eaf1*-MO, *eaf2*-MO and *eaf1* + *eaf2*-MO at 24 hpf, and the quantification of the WISH data **(A9, A10). (B, C)** WISH analysis of *hbbe3* (**B1**-**B8**) and *gata1a* (**C1**-**C8**) in the *eaf1^-/-^*, *eaf2^-/-^*, and WT embryos, and the corresponding groups injected with *eaf1* mRNA and *eaf2* mRNA, respectively, and quantification of the WISH data of *hbbe3* (**B9**, **B10**) and *gata1a* (**C9**, **C10**). Each experiment was repeated three times, and a representative result is shown. All embryos are shown in lateral view, anterior to the left. Data are presented as mean ± SD. **P* < 0.05, ***P* < 0.01, ****P* < 0.001, NS, not significant. Scale bars = 200 μm.


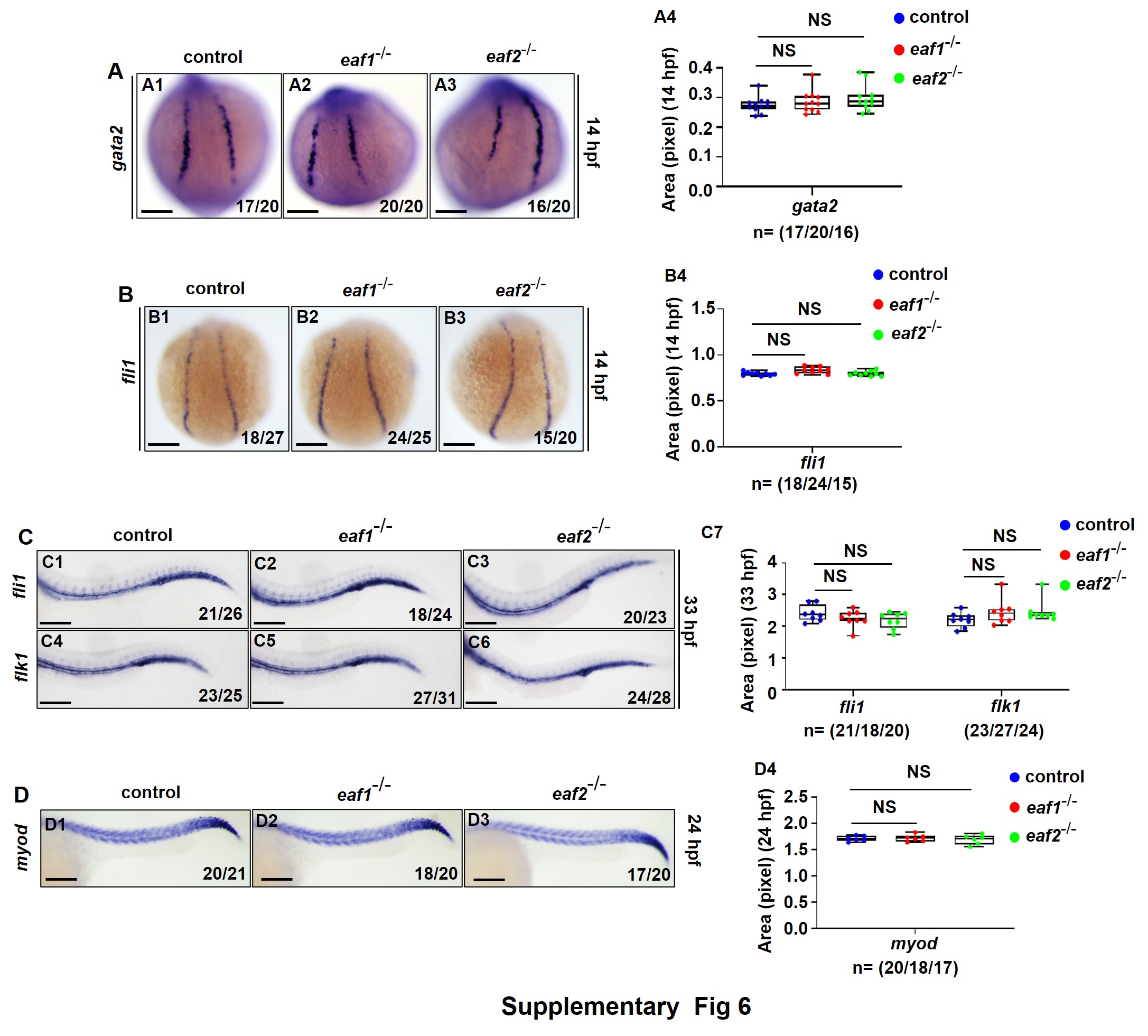


**Fig. S6** **Effects of *eaf1/2* deficiency on the expression of genes *gata2/fli1/flk1/myod.***

**(A)** WISH analysis of the expression of the gene *gata2* in *eaf1*^-/-^, *eaf2*^-/-^, and WT embryos at 14 hpf **(A1-A3)**, and quantitative analysis of the WISH data **(A4). (B)** WISH analysis of the expression of gene *fli1* in *eaf1*^-/-^, *eaf2*^-/-^, and WT embryos at 14 hpf **(B1-B3)**, and quantitative analysis of the WISH data **(B4). (C)** WISH analysis of the expressions of genes *fli1* and *flk1* in *eaf1*^-/-^, *eaf2*^-/-^, and WT embryos at 33 hpf **(C1-C6)**, and quantitative analysis of their respective WISH data **(C7). (D)** WISH analysis of the expression of gene *myod* in *eaf1*^-/-^, *eaf2*^-/-^, and WT embryos at 24 hpf **(D1-D3),** and quantitative analysis of the WISH data **(D4).** Each experiment was repeated at least three times, with similar results for two or three replicates, and a representative result was shown. Data are mean ± SD. **P* < .05, ***P* < .01, ****P* < .001. NS, not significant. Scale bar = 75 μm (**A1-A3, B1-B3**) and 250 μm (**C1-C6, D1-D3**).


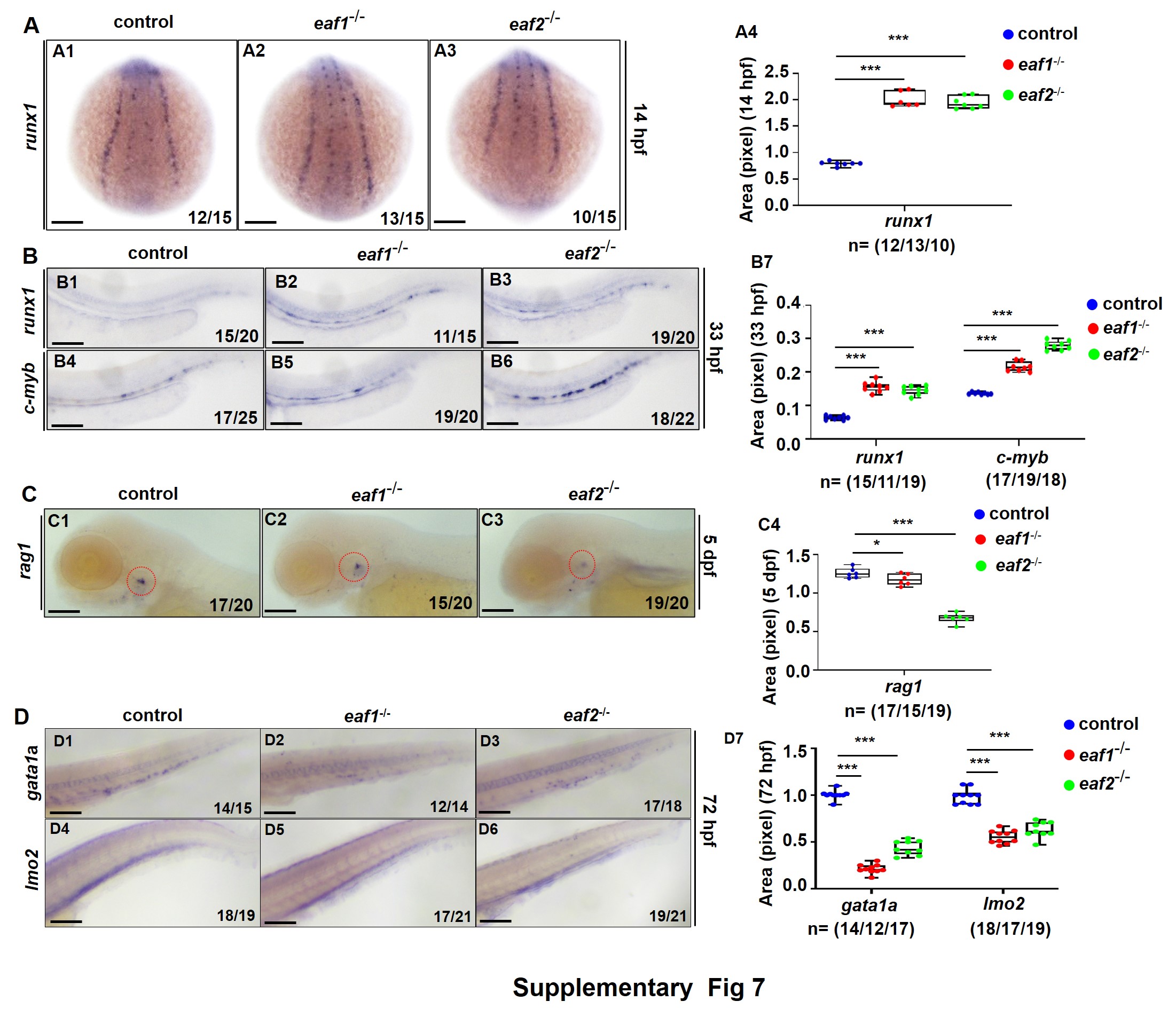


**Fig. S7 Effects of *eaf1/2* deficiency on the expression of *runx1, c-myb*, *rag1, gata1a* and *lmo2.* (A)** WISH analysis of the expression of gene *runx1* in *eaf1*^-/-^, *eaf2*^-/-^, and WT embryos at 14 hpf **(A1-A3),** and quantitative analysis of the WISH data **(A4). (B)** WISH analysis of the expression of genes *runx1* and *c-myb* in *eaf1*^-/-^, *eaf2*^-/-^, and WT embryos at 33 hpf **(B1-B6)**, and quantitative analysis of the WISH data **(B7).** **(C)** WISH analysis of the expression of gene *rag1* in *eaf1*^-/-^, *eaf2*^-/-^, and WT embryos at 5 dpf **(C1-C3)**, and quantitative analysis of the WISH data **(C4). (D)** WISH analysis of the expression of gene *gata1a* and *lmo2* in *eaf1*^-/-^, *eaf2*^-/-^, and WT embryos at 72 hpf **(D1-D6)**, and quantitative analysis of the WISH data **(D7).** Each experiment was repeated at least three times, with similar results for two or three replicates, and a representative result was shown. Data are mean ± SD. **P* < .05, ***P* < .01, ****P* < .001. NS, not significant. Scale bar= 75 μm (**A1-A3, C1-C3 and D1-D6**), 250 μm (**B1-B6**).


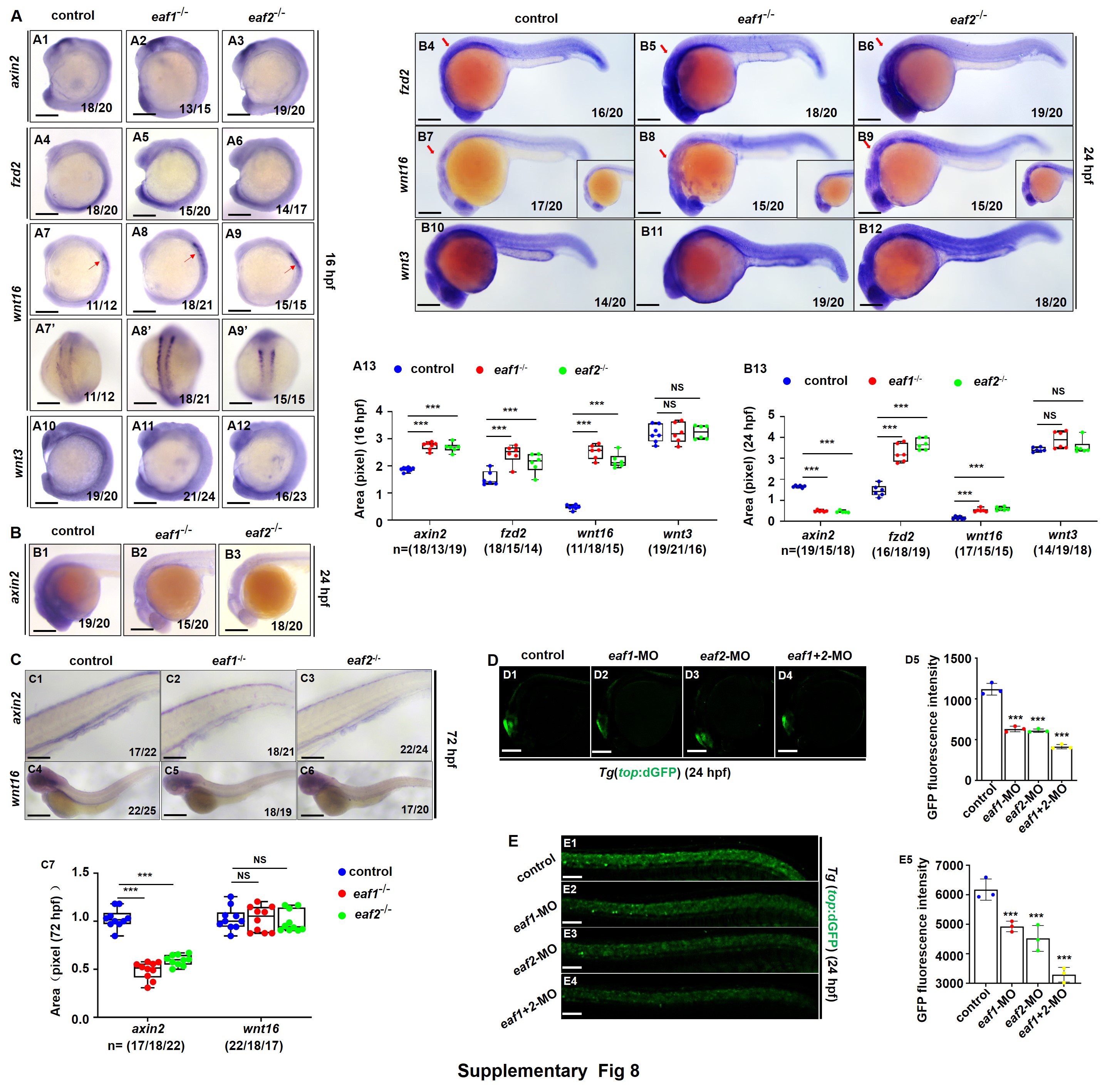


**Fig. S8 Effects of *eaf1/2* deficiency on WNT/β-catenin signaling during fish embryogenesis. (A)** WISH analysis of the expression of WNT/β-catenin signaling indicator *axin2* and WNT/β-catenin signaling factors *fzd2, wnt16,* and *wnt3* in *eaf1*^-/-^, *eaf2*^-/-^, and WT embryos at 16 hpf **(A1-A12)**, and statistical analysis of the staining results **(A13)**. **(B)** WISH analysis of the expression of WNT/β-catenin signaling indicator *axin2* (**B1-B3**) as well as *fzd2, wnt16,* and *wnt3* in *eaf1*^-/-^, *eaf2*^-/-^ and WT embryos at 24 hpf **(B4-B12)**, and statistical analysis of the staining results **(B13)**. **(C)** WISH analysis of the expression of gene *axin2* and *wnt16* in *eaf1*^-/-^, *eaf2*^-/-^, and WT embryos at 72 hpf **(C1-C6)**, and quantitative analysis of the WISH data **(C7). (D, E)** Top GFP expression in the brain and spinal cord in the *Tg* (*top*: gfp) embryos injected with *eaf1*-MO, *eaf2*-MO and *eaf1-*MO plus *eaf2-*MO, respectively. **A1**-**A12**, **B1**-**B12**, **C1-C6, D1-D4, E1-E4,** lateral view, anterior to the left; **A7’**- **A9’**, dorsal view, anterior to the up. **P* < .05, ***P* < .01, ****P* < .001. NS, not significant. Scale bar= 75 μm (**A1-A12, A7’**-**A9’, B1-B3, C1-C3 and D1-D4**), 100 μm **(E1-E4)** and 250 μm (**C4-C6, B4-B12**)**.**


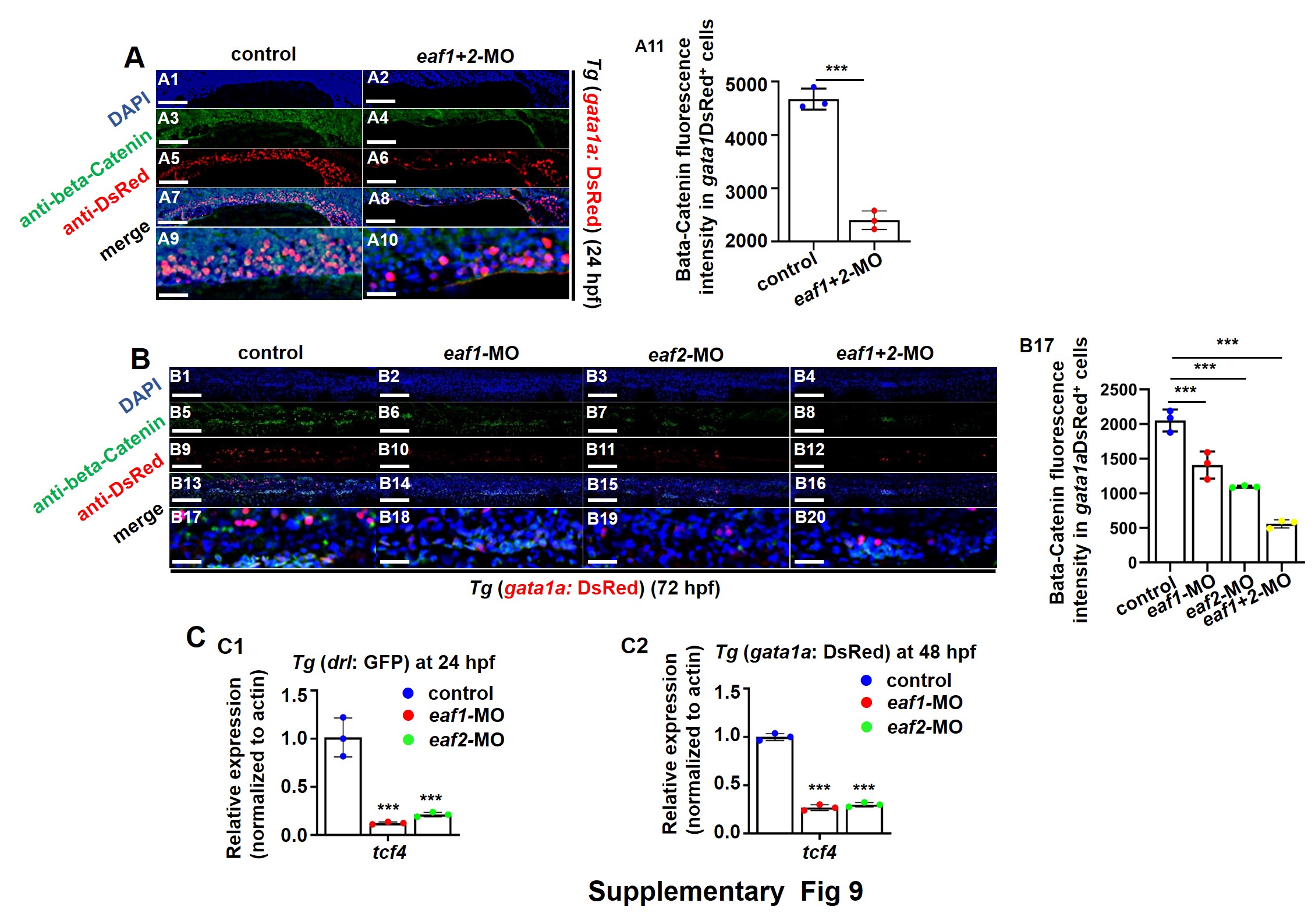


**Fig. S9 Immunofluorescence of β-Catenin protein in RBCs (*gata1a*^+^ cells). (A)** Double staining of *gata1a*DsRed^+^ and β-catenin in the control and embryos injected with *eaf1*-MO plus *eaf2*-MO at 24 hpf (**A1-A10)**, and quantification of β-Catenin immunofluorescence intensities in *gata1a*DsRed^+^ (**A11**), and A9, A10 show the magnified views of A7, A8, respectively. **(B)** Double staining of *gata1a*DsRed^+^ and β-catenin in the control and embryos injected with *eaf1*-MO, *eaf1*-MO, and *eaf1*-MO plus *eaf2*-MO at 72 hpf (**B1-B20)**, and quantification of β-catenin immunofluorescence intensities in *gata1a*DsRed^+^ (**B21**), and B17-B20 show the magnified views of B13-B16, respectively. Each experiment was repeated at least three times, with similar results for two or three replicates, and a representative result was shown. All embryos are shown in lateral view, anterior to the left. Data are presented as mean ± SD. **P* < 0.05, ***P* < 0.01, ****P* < 0.001, NS, not significant. Scale bars = 100 μm (**A1-A8 and B1-B16**) and 50 μm **(A9-A10, B17-B20).**


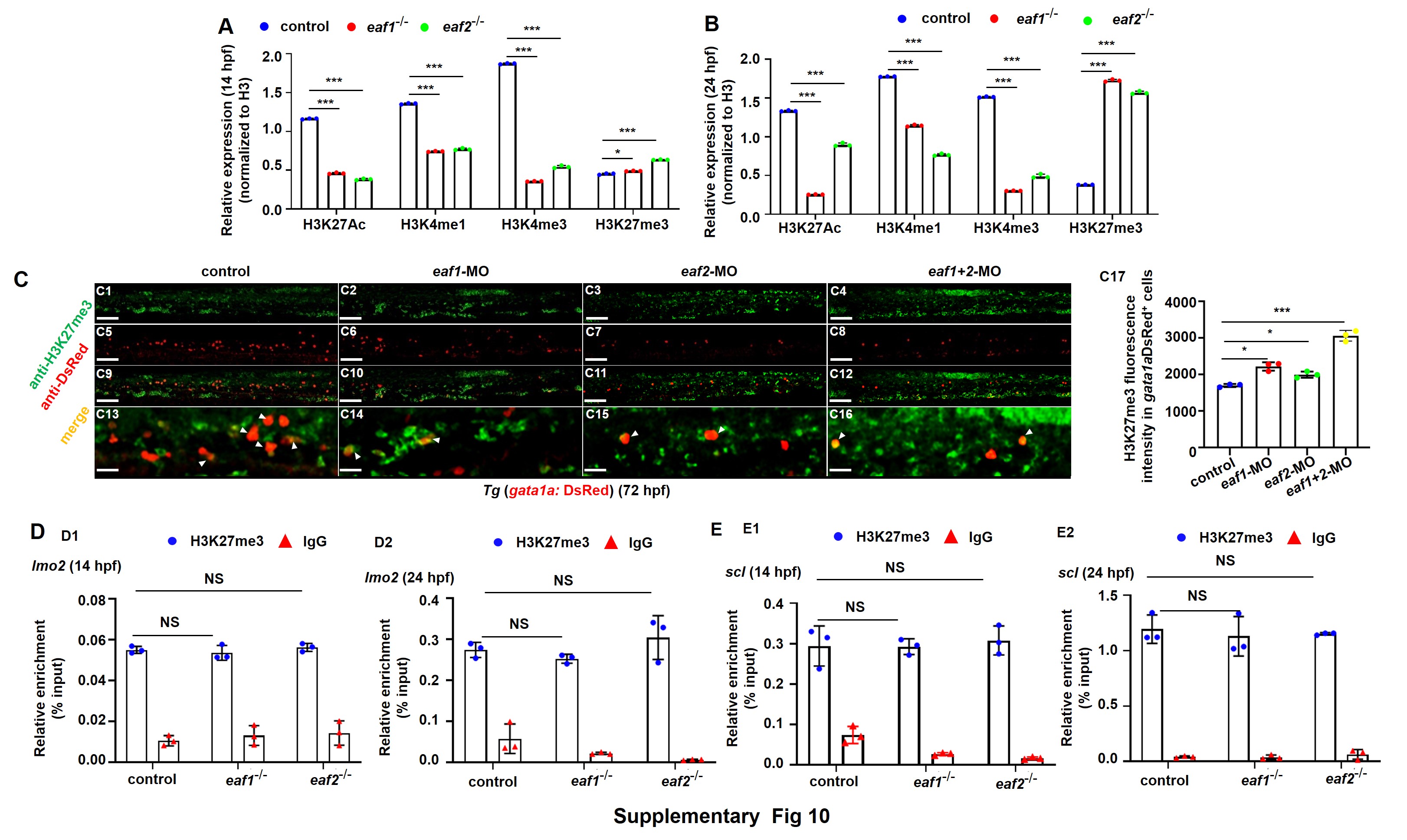


**Fig. S10 Effects of *eaf1/2* deficiency on the protein levels of H3K27ac, H3K4me1, H3K4me3, and H3K27me3. (A, B)** Quantitative analysis of protein level of H3K27ac, H3K4me1, H3K4me3, and H3K27me3 in *eaf1*^-/-^, *eaf2*^-/-^, and WT embryos at 14 hpf and 24 hpf respectively. **(C)** Double staining of *gata1a*DsRed^+^ and H3K27me3, in the control and embryos injected with *eaf1*-MO, *eaf2*-MO, and *eaf1*-MO plus *eaf2*-MO at 72 hpf (**C1-C16)**, and quantification of H3K27me3 immunofluorescence intensities in *gata1a*DsRed^+^ (**C17**), and C13-C16 show the magnified views of C9-C12, respectively. **(D, E)** Chromatin immunoprecipitation-qPCR (ChIP-qPCR) analysis of the binding enrichment of protein H3K27me3 on the promoter of *lmo2* **(D1-D2)** and *scl* **(E1-E2)** in *eaf1*^-/-^, *eaf2*^-/-^, and WT embryonic cells at both 14 hpf and 24 hpf, with anti- IgG used as a negative control. Each experiment was repeated at least three times, with similar results for two or three replicates, and a representative result was shown. All embryos are shown in lateral view, anterior to the left. Data are presented as mean ± SD. **P* < 0.05, ***P* < 0.01, ****P* < 0.001, NS, not significant. Scale bars = 100 μm.


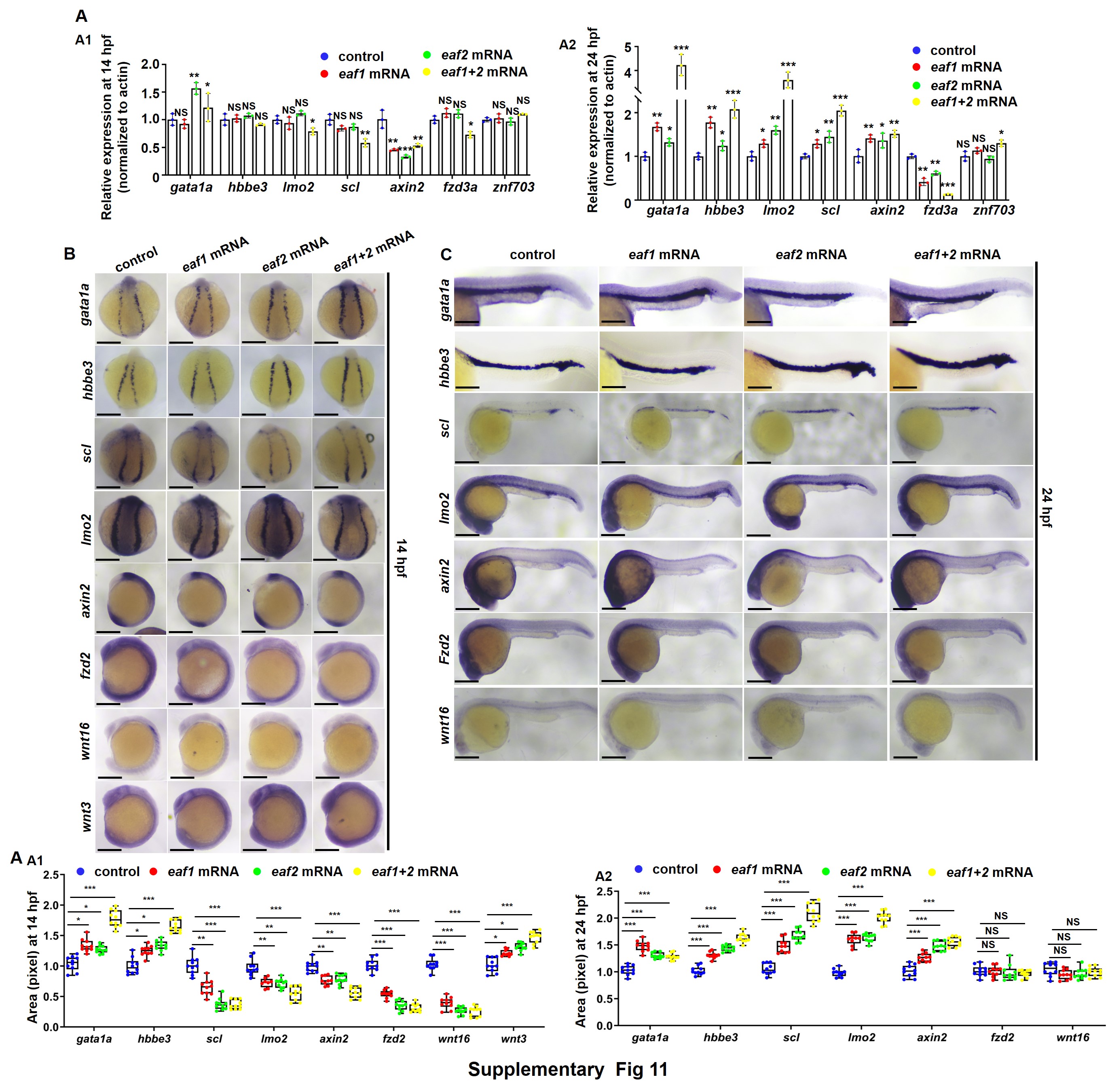


**Fig. S11 Effects of overexpression of *eaf1*, *eaf2* and overexpression of both genes on erythrogenesis and Wnt signaling. (A)** qRT–PCR expression analysis of *gata1a,* *hbbe3*, *scl*, *lmo2*, *axin2*, *fzd3a* and *znf703* in the control and embryos injected with *eaf1* mRNA, *eaf2* mRNA, *eaf1* mRNA plus *eaf2* mRNA at 14 hpf **(A1)** and 24hpf **(A2)**, respectively. **(B, C)** WISH analysis of the expression of *gata1a,* *hbbe3*, *scl*, *lmo2*, *axin2*, *fzd2, wnt16,* and *wnt3* the control and embryos injected with *eaf1* mRNA, *eaf2* mRNA, *eaf1* mRNA plus *eaf2* mRNA at 14 hpf **(B1)** or 24hpf **(C1)**, and quantitative analysis of expression of *gata1a*, *hbbe3*, *scl*, *lmo2*, *axin2*, *fzd2*, *wnt16*, and *wnt3* at 14 hpf **(B2)** and 24 hpf **(C2)**. Data are presented as mean ± SD. **P* < 0.05, ***P* < 0.01, ****P* < 0.001, NS, not significant.


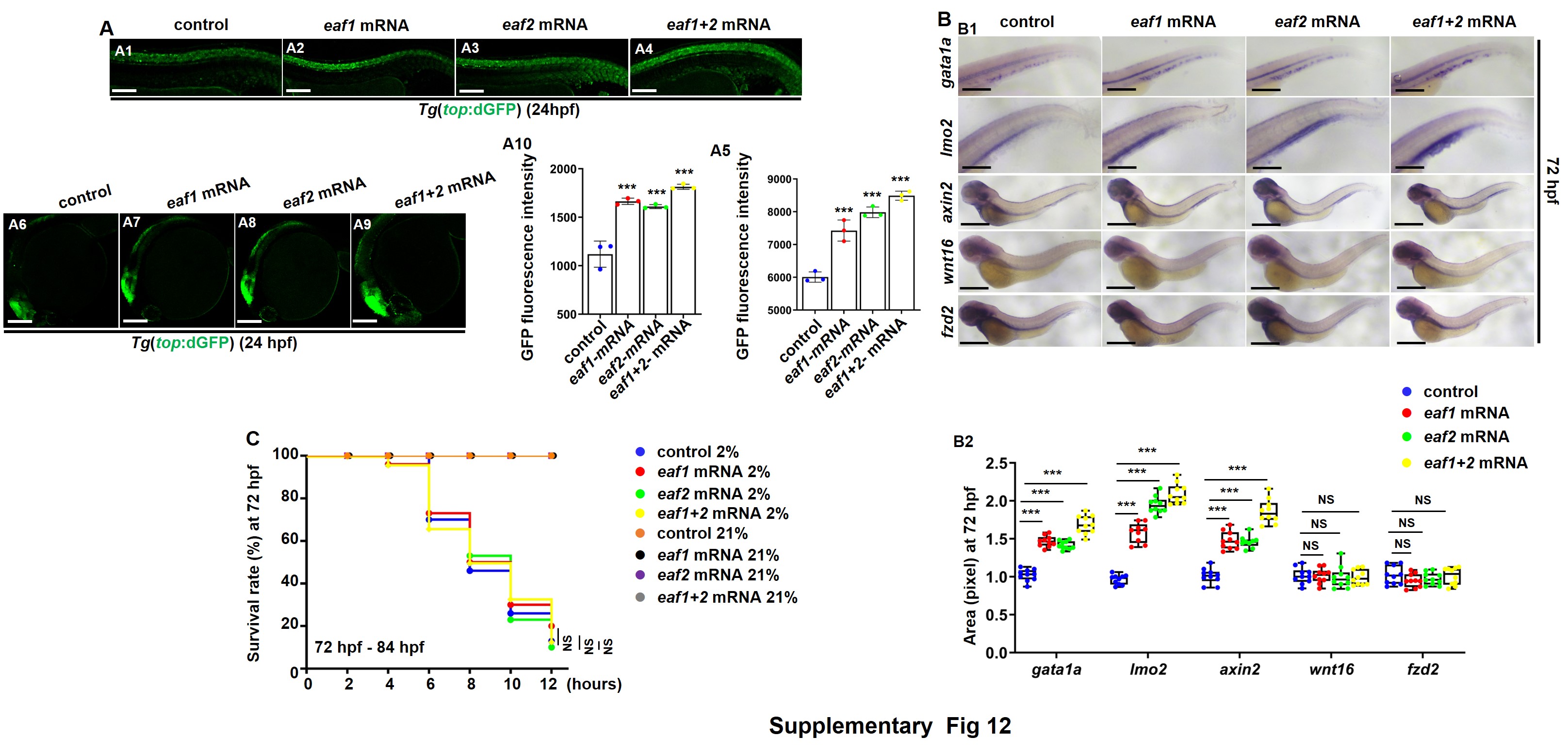


**Fig. S12 Effects of overexpression of *eaf1*, *eaf2* and overexpression of both genes on expression of *gata1a*, *lmo2*, *axin2*, *wnt16* and *fzd2*, and the** [**hypoxic tolerance**](https://dict.youdao.com/w/hypoxic%20tolerance/#keyfrom=E2Ctranslation) **of the larvae with ectopic expression. (A)** Top GFP expression in the brain and spinal cord in the *Tg* (*top*: dGFP) embryos injected with *eaf1* mRNA, *eaf2* mRNA, *eaf1* mRNA plus *eaf2* mRNA, respectively. **(B)** WISH analysis of *gata1a*, *lmo2*, *wnt16,* and *fzd2* in the control and embryos injected with *eaf1* mRNA, *eaf2* mRNA, *eaf1* mRNA plus *eaf2* mRNA at 72 hpf, and quantitative analysis of expression of *gata1a*, *lmo2*, *axin2*, *wnt16*, *fzd2*. **(C)** The survival rate curves of the embryos (the control and embryos injected with *eaf1* mRNA, *eaf2* mRNA, *eaf1* mRNA plus *eaf2* mRNA) exposed to normoxia (21% O_2_) or hypoxia (2% O_2_) beginning at 72 hpf for 12 h. Each experiment was repeated at least three times, with similar results for two or three replicates, and a representative result was shown. All embryos are shown in lateral view, anterior to the left. Data are presented as mean ± SD. **P* < 0.05, ***P* < 0.01, ****P* < 0.001, NS, not significant.

| **Supplementary Table 1: Genes tested in this study** | |  |
| --- | --- | --- |
| **Gene symbol** | **Full names** |  |
|  |  |  |
| *axin2* | conductin, axil |  |
| *cited2* | Cbp/p300-interacting transactivator, with Glu/Asp-rich carboxy-terminal domain, 2 |  |
| *c-myb* | v-myb avian myeloblastosis viral oncogene homolog |  |
| *drl* | draculin |  |
| *eaf1* | ELL associated factor 1 |  |
| *eaf2* | ELL associated factor 2 |  |
| *fli1* | Fli-1 proto-oncogene, ETS transcription factor |  |
| *flk1* | kinase insert domain receptor like |  |
| *fzd2* | frizzled class receptor 2 |  |
| *fzd3a* | frizzled class receptor 3a |  |
| *gata1a* | GATA binding protein 1a |  |
| *gata2* | GATA binding protein 2a |  |
| *hbbe1* | hemoglobin beta embryonic-1.1 |  |
| *hbbe2* | hemoglobin beta embryonic-2 |  |
| *hbbe3* | hemoglobin beta embryonic-3 |  |
| *hif1αb* | hypoxia inducible factor 1 subunit alpha b |  |
| *hif2αb/epas1b* | endothelial PAS domain protein 1b |  |
| *hif3α/hif1al* | hypoxia inducible factor 1 subunit alpha, like |  |
| *ldha* | ldha lactate dehydrogenase A4 |  |
| *lmo2* | LIM domain only 2 (rhombotin-like 1) |  |
| *myod* | myogenic differentiation |  |
| *olig2* | oligodendrocyte lineage transcription factor 2 |  |
| *pai1/serpine1* | serpin peptidase inhibitor, clade E (nexin, plasminogen activator inhibitor type 1), member 1 |  |
| *rag1* | recombination activating 1 |  |
| *runx1* | RUNX family transcription factor 1 |  |
| *scl* | T-cell acute lymphocytic leukemia 1 |  |
| *tcf4* | transcription factor 4 |  |
| *wnt16* | wingless-type MMTV integration site family, member 16 |  |
| *wnt3wnt16* | wingless-type MMTV integration site family, member 3wingless-type MMTV integration site family, member 16 |  |
| *znf703wnt3* | zinc finger protein 703wingless-type MMTV integration site family, member 3 |  |
| *znf703* | zinc finger protein 703 |  |

| **Supplementary Table 2: Sequences of primers for mutated target loci detection** | |
| --- | --- |
| **Primer** | **Sequence** |
| *eaf1*-F-291bp | 5' GCGTCTTCTCCTTTTGTCT 3' |
| *eaf1*-R-291bp | 5' TGCTTGGTAATATGCTCAG 3' |
| *eaf2*-F-179bp | 5' GGCTGAGGGAAGCAGTAAGA 3' |
| *eaf2*-R-179bp | 5' GACCAGACATGTTACCTCGC 3' |

| **Supplementary Table 3: Sequences of primers for RT-qPCR and One Step Cell-Direct qRT–PCR** | |
| --- | --- |
| **Primer** | **Seuqence** |
| *axin2*-RT-F | 5' CCTCCCAACACCTTAGCAC 3' |
| *axin2*-RT-R | 5' TTTCATCATCCGCCGATAT 3' |
| *cited2*-RT-F | 5' GTTCCGAGACAGTATCGCTAAG 3' |
| *cited2*-RT-R | 5' ATCAAGACCTCCTCGTCAATAA 3' |
| *eaf1*-F-123bp | 5' GGAAACACATCTCTCGCCCA 3' |
| *eaf1*-R-123bp | 5' CTGTCACTGCCCGATTCACT 3' |
| *eaf2*-F-123bp | 5' CACGTCCTCAAACTAGGCGA 3' |
| *eaf2*-R-123bp | 5' TTTTCCCACTTCCAGCTCCC 3' |
| *fzd3a-*RT-F | 5' GTGTTTTGGGTCGGGAGT 3' |
| *fzd3a-*RT-R | 5' GGAGCTGGGTAAAGTCGG 3' |
| *gata1a*-F-152bp | 5' CCAGAGTTTGGGTGATGGGT 3' |
| *gata1a*-R-152bp | 5' CTGTCACTGCCCGATTCACT 3' |
| *hbbe3*-F-101bp | 5' ATGCTTGGTCGTCTATCCGT 3' |
| *hbbe3*-R-101bp | 5' CGTGCGCTTTGACTTTTGGG 3' |
| *hif1αb*-RT-F | 5' ACTTCCCTTTCAGCTCTGCC 3' |
| *hif1αb*-RT-R | 5' CCGTAATCCATGGGGCTGTT 3' |
| *hif2αb*-RT-F | 5' TCACAAGCCTCTTCCAACCC 3' |
| *hif2αb*-RT-R | 5' GGCGGGATCATGGTATGGAG 3' |
| *hif3α*-RT-F | 5'AAATCCGAGACTCCCACCGA 3' |
| *hif3α*-RT-R | 5' TTGGTCGCAGGGATGAACAA 3' |
| *gata1a*-F-152bp | 5' CCAGAGTTTGGGTGATGGGT 3' |
| *gata1a*-R-152bp | 5' CTGTCACTGCCCGATTCACT 3' |
| *ldha*-RT-F | 5' CCTTCTCAAGGATCTGACCG 3' |
| *ldha*-RT-R | 5' ACACTGTAATCTTTATCCGC 3' |
| *myod*-F-133bp | 5' TATCCCCTTCCCCATCCC 3' |
| *myod*-R-133bp | 5' CCTTGCTCAGTCGCCTCC 3' |
| *olig2*-F-150bp | 5' CGCTGCTGTCTCGCATTCC 3' |
| *olig2*-R-150bp | 5' ACGGAGGCACCTGGCACAT 3' |
| *pai1*-RT-F | 5' ATTCCAAGGTTCTCCATGGA 3' |
| *pai1*-RT-R | 5' GGTTCCTCAGTAGTAATGCG 3' |
| *tcf4*-F-150bp | 5' TCCTGGGTTGCCTTCATCAG 3' |
| *tcf4*-R-150bp | 5' TGTTGTATCCGGGCTGGTTC 3' |
| *znf703*-RT-F | 5' GCCCGTGAGCATTGAG 3' |
| *znf703*-RT-R | 5' TCGGCATTCTGATCCAG 3' |
| *znf703*-RT-F | 5' GCCCGTGAGCATTGAG 3' |
| *znf703*-RT-R | 5' TCGGCATTCTGATCCAG 3' |

| **Supplementary Table 4: Sequences of primers for full-length CDS** | |
| --- | --- |
| **Primer** | **Sequence** |
| *eaf1*-F-864bp | 5' TAATACGACTCACTATAGGGATGAACGGCAGCTCGAACC 3' |
| *eaf1*-R-864bp | 5' TCAGTCGATGTCGCTGTCACTGC 3' |
| *eaf2*-F-877bp | 5' TAATACGACTCACTATAGGGTGGATTAGAATGAATGGAACAGC 3' |
| *eaf2*-R-877bp | 5' TCAGTCATCATCGCTTTCACTTCCA 3' |

| **Supplementary Table 5: Primer pairs for WNT Signaling genes examined in the study** | | |  |
| --- | --- | --- | --- |
| **Gene** | **Primer sequence (5'to3')** | **GenBank ID** |  |
|  |  |  |  |
| *wnt3* | F：5' TGAGGCAGGACGAATGACCA 3' | NC_007123.7 |  |
|  | R：5' TAATACGACTCACTATAGGGTTTGCGCTTTTCTGTCCGTG 3' |  |  |
| *wnt16* | F：5' AGGGAAGTTGGATGTGGTTGG 3' | NC_007115.7 |  |
|  | R：5' TAATACGACTCACTATAGGGGGGTGACTGCATGAACCAGG 3' |  |  |
| *fzd2* | F：5' AGACGTGCTAAGCGGAGTTT 3' | NC_007114.7 |  |
|  | R：5' TAATACGACTCACTATAGGGAGTGCAGTGTCTTTCCCGAC 3' |  |  |

| **Supplementary Table 6: sequences of primer used for ChIP-qPCR** | | |  |
| --- | --- | --- | --- |
| **Gene** | **Primer sequence (5'to3')** | **GenBank ID** |  |
|  |  |  |  |
| *scl*(TCF4) | F: 5' TTTATAGATGGACATTGGGTA 3' | NM_213237.1 |  |
|  | R: 5' TGAGTTGCGATTGTGCTA 3' |  |  |
| *scl*(H3K27me3) | F: 5' ATAGCACAATCGCAACTC 3' |  |  |
|  | R: 5' CCTTTACCGCATGACTT 3' |  |  |
| *lmo2*(TCF4/H3K27me3) | F: 5' CTGACTGACTCCGTGTTT 3' | NM_131111.1 |  |
|  | R: 5' GTCCCGTTATTGTAGGG 3' |  |  |
| *gata1a*(H3K27me3) | F: 5' GTCTATAAGGTCATATAGGC 3' | NM_131234.1 |  |
|  | R: 5' CTTCAGTCTTTGGGAACTAG 3' |  |  |
